# Developing a universal multi-epitope protein vaccine candidate for enhanced borna virus pandemic preparedness

**DOI:** 10.3389/fimmu.2024.1427677

**Published:** 2024-12-05

**Authors:** Jingjing Zhang, Youfang Yang, Binyu Wang, Wanting Qiu, Helin Zhang, Yuyang Qiu, Jing Yuan, Rong Dong, Yan Zha

**Affiliations:** ^1^ School of Basic Medicine, Guangzhou Medical University, Guangzhou, China; ^2^ Department of Nephrology, Guizhou Provincial People's Hospital, Guiyang, China; ^3^ NHC Key Laboratory of Pulmonary Immunological Diseases, Guizhou Provincial People's Hospital, Guiyang, China; ^4^ School of Clinical Medicine, Guizhou Medical University, Guiyang, China; ^5^ Department of Nephrology, The First Clinical Institute, Zunyi Medical University, Zunyi, China; ^6^ School of Medicine, Guizhou University, Guiyang, China

**Keywords:** Borna virus, immunoinformatics, epitopes, vaccine, molecular docking, molecular dynamics simulation

## Abstract

**Introduction:**

Borna disease virus 1 (BoDV-1) is an emerging zoonotic RNA virus that can cause severe acute encephalitis with high mortality. Currently, there are no effective countermeasures, and the potential risk of a future outbreak requires urgent attention. To address this challenge, the complete genome sequence of BoDV-1 was utilized, and immunoinformatics was applied to identify antigenic peptides suitable for vaccine development.

**Methods:**

Immunoinformatics and antigenicity-focused protein screening were employed to predict B-cell linear epitopes, B-cell conformational epitopes, and cytotoxic T lymphocyte (CTL) epitopes. Only overlapping epitopes with antigenicity greater than 1 and non-toxic, non-allergenic properties were selected for subsequent vaccine construction. The epitopes were linked using GPGPG linkers, incorporating β-defensins at the N-terminus to enhance immune response, and incorporating Hit-6 at the C-terminus to improve protein solubility and aid in protein purification. Computational tools were used to predict the immunogenicity, physicochemical properties, and structural stability of the vaccine. Molecular docking was performed to predict the stability and dynamics of the vaccine in complex with Toll-like receptor 4 (TLR-4) and major histocompatibility complex I (MHC I) receptors. The vaccine construct was cloned through in silico restriction to create a plasmid for expression in a suitable host.

**Results:**

Among the six BoDV-1 proteins analyzed, five exhibited high antigenicity scores. From these, eight non-toxic, non-allergenic overlapping epitopes with antigenicity scores greater than 1 were selected for vaccine development. Computational predictions indicated favorable immunogenicity, physicochemical properties, and structural stability. Molecular docking analysis showed that the vaccine remained stable in complex with TLR-4 and MHC I receptors, suggesting strong potential for immune recognition. A plasmid construct was successfully generated, providing a foundation for the experimental validation of vaccines in future pandemic scenarios.

**Discussion:**

These findings demonstrate the potential of the immunoinformatics-designed multi-epitope vaccines for the prevention and treatment of BoDV-1. Relevant preparations were made in advance for possible future outbreaks and could be quickly utilized for experimental verification.

## 1 Introduction

Borna virus type 1 (BoDV-1) is a non-segmented, negative-strand RNA virus with a case fatality rate of up to 90% (1). Epidemiological data from 1999 to 2019 indicated a 77.78% positivity rate of BoDV-1 RNA in patients with fatal encephalitis at a diagnostic center (2). Since 2018, upon confirmation of BoDV-1’s zoonotic transmission, retrospective studies of related cases have reported a mortality rate as high as 94% (3). The virus’s extreme lethality is largely attributed to its ability to replicate intracellularly after entry into the central nervous system via the olfactory pathway, which triggers a strong immune response (4–6). Clinical manifestations include headache, fever, and loss of consciousness, which progress to neurological symptoms, deep coma, extensive involvement of the brain stem, and ultimately to highly fatal encephalitis (7).

Although BoDV-1 exhibits a high fatality rate, it was not until 2018 that two clinical cases of fatal BoDV-1 in solid organ transplant recipients confirmed its zoonotic potential, prompting increased monitoring efforts (8). In March 2020, Germany mandated direct pathogen detection for Borna viruses, including BoDV-1, in human samples (9). Since then, the number of confirmed cases of BoDV-1 encephalitis has gradually increased, and some evidence of mutations associated with pandemic potential was found. Recent findings indicated the virus’s development of a specific immune escape mechanism in some natural hosts (3). With the proliferation of related viruses such as BoDV-2 and VSV-1 in birds and reptiles, concerns arose regarding a potential increase in related mammalian viruses, leading to more severe outbreaks (10, 11). In the event of a large-scale outbreak, the high mortality and severe symptoms associated with BoDV-1 infection could pose a significant public health challenge. Consequently, urgent efforts are required to intensify research on BoDV-1 to devise effective prevention and control strategies in anticipation of a potential pandemic.

However, a standardized BoDV-1 infection management program remains due to research constraints and reliance on retrospective studies, leaving our understanding of the virus’s transmission and disease mechanism insufficient (12). At the same time, the development of a vaccine against Borna disease virus 1 (BoDV-1) remains a formidable challenge due to the time and expense associated with traditional vaccine development methods (13).

Based on this, immunoinformatics was selected for vaccine design. Immunoinformatics technology revolutionizes vaccine development timelines by streamlining candidate selection through epitope-based design (14). This approach accelerates development, reduces experimental costs, and enhances safety by circumventing infectivity linked to conventional whole-pathogen vaccines (15). A notable example was Moderna’s mRNA-1273 vaccine, which reached its first human phase I clinical trial within just 66 days after the SARS-CoV-2 genome sequence was published, highlighting the efficacy of this innovative methodology (16).

At the same time, the research and development of immunoinformatics for various pathogens is developing rapidly. Currently, vaccines against group B streptococcus and Brucella have shown good immune effects in mice (17, 18). Moreover, with the proliferation of publicly accessible databases and the exponential enhancement of computational processing capabilities, coupled with the advancing sophistication of biological data analysis software, the reliability and robustness of immunoinformatics technology continue to escalate (19). This targeted approach holds the promise of substantially truncating the development timeline for combating BoDV-1, a virus renowned for its lethality and elusiveness.

In this investigation, immunogenic epitopes were discerned from the comprehensive genome sequence of BoDV-1 utilizing bioinformatic screening techniques. To provoke a heightened immune response, a series of B-cell and T-cell epitopes were carefully selected and subsequently used for safety assessment following overlapping screening. The formulated multi-epitope vaccine construct underwent thorough evaluation regarding potential allergenicity, immunogenicity, physicochemical stability, and its capacity to induce immune responses. Rapid advancements in computer-based vaccine design provide a critical framework for pandemic preparedness, enabling swift response capabilities for potential BoDV-1 outbreaks.

## 2 Materials and methods

**Figure 1:**
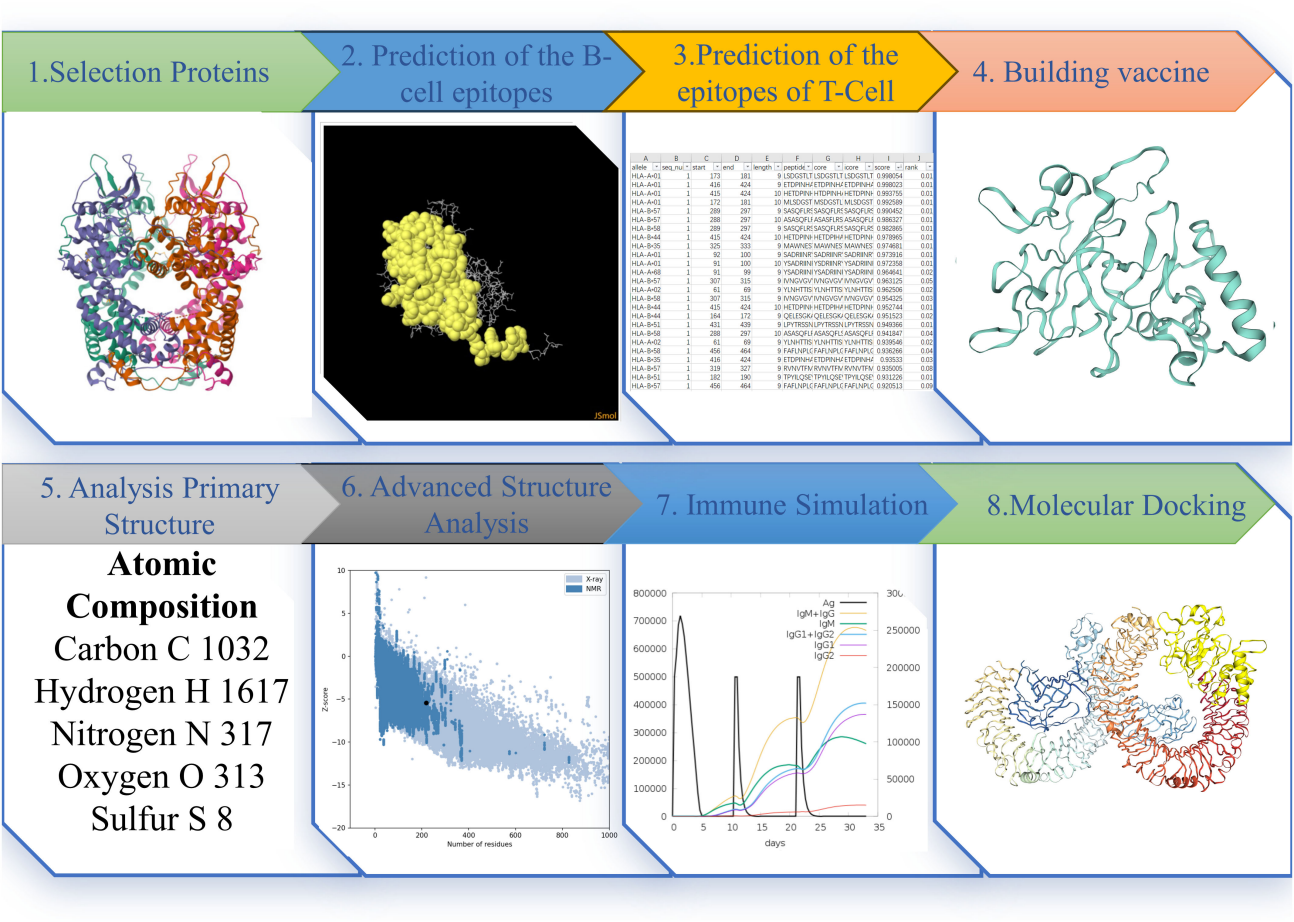
The Figure illustrates the pertinent research methods and sequences employed in this study. Each step’s depiction represents the results obtained at that particular stage.

The procedural outline of the current study was concisely elaborated upon in **Figure 1**.

### 2.1 Protein selection

Initially, the entire genomic protein sequence of Borna Disease Virus 1 (BoDV-1) was extracted from the National Center for Biotechnology Information (NCBI) database (http://www.ncbi.nlm.nih.gov). To enhance the immune response of the vaccine, screening was conducted for proteins with high antigenicity for further vaccine development. To assess its antigenicity, the VaxiJenv2.0 tool was used to predict the antigenicity of the downloaded protein sequence (https://www.ddg-pharmfac.net/vaxijen/VaxiJen/VaxiJen.html). This tool employs a model specifically developed for entire proteins, utilizing a viral protein dataset and achieving a prediction accuracy ranging from 70% to 89%. The predictive performance of the model was evaluated using leave-one-out cross-validation (LOO-CV) and external validation (20). A threshold of 0.4 was set for antigenicity scores; proteins scoring above 0.4 were deemed potential antigens and were selected for further analysis, particularly epitope prediction (21). The selected protein sequence served as the reference. This reference sequence was subsequently used to identify specific antigenic peptides in downstream analyses.

### 2.2 Prediction of the B-cell epitopes

B-cell epitopes are specific regions or sites on antigen molecules that bind selectively to the B-cell receptor (BCR), thereby activating B-cells and triggering antibody production. This antibody production is essential for both initiating and modulating immune responses (22). Epitopes are of high interest in vaccine design, as they offer an alternative to whole antigens and can stimulate targeted antibody responses through immune system activation (23). Depending on the amino acid sequence continuity, B-cell epitopes were categorized into linear and conformational epitopes.

#### 2.2.1 Linear epitopes

The computational tool Immune Epitope Database (IEDB) server (http://tools.iedb.org/bcell/) was employed to predict linear B-cell epitopes. The amino acid sequence of the selected protein was entered into the server, and the BepiPred-2.0 was used for the prediction of continuous B-cell epitopes. BepiPred-2.0 employs a random forest algorithm trained on epitopes derived from protein structures of antibody-antigen complexes (24). The resulting epitope predictions can be displayed in tabular form. The linear B-cell antigen peptides obtained will be used for subsequent co-localization with non-linear antigen peptides.

#### 2.2.2 Tertiary structure and nonlinear epitopes prediction

The recognition and binding of nonadjacent amino acid residues on an antigen molecule by the B-cell receptor (BCR) result in the formation of a conformationally nonlinear epitope, which subsequently triggers a specific antibody response (25). To accurately predict B-cell nonlinear epitopes, it is essential to account for the protein’s three-dimensional (3D) structure. The PBD format file of the three-dimensional (3D) protein structure was obtained from the UniProt database (https://www.uniprot.org). If a 3D structure was not available in UniProt, prediction tools trRosetta (https://yanglab.qd.sdu.edu.cn/trRosetta/) were utilized as alternatives. It was based on direct energy minimization and utilized constrained Rosetta to construct protein structures (26). Finally, the Structure Assessment tool of the SwissModel platform (https://swissmodel.expasy.org/assess) was used to evaluate the protein’s three-dimensional structure (27).

Subsequent to this, the predictive structure obtained from each server was then optimized through the Galaxy server (https://galaxy.seoklab.org/cgi-bin/submit.cgi?type=REFINE2). In the server, GalaxyRefine was selected for optimization, which adopts an iterative optimization method to optimize the inaccurate local regions and the overall protein structure (28). The PDB files were then submitted to Ellipro (http://tools.iedb.org/ellipro/) for nonlinear epitope prediction. Ellipro identified potential nonlinear (conformational) epitopes by calculating exposed areas and surface accessibility on the protein surface (29). Finally, the linear and nonlinear epitopes of the predicted protein were examined for overlapping sequences.

### 2.3 Prediction of the epitopes of T-Cell

Cellular immune responses are orchestrated through the recognition of cell MHC I epitopes, triggering the activation of CD8+ T-cells and conferring protection against viral infections. BoDV-1, as a linear negative-sense single stranded RNA virus, penetrates host cells, thus prompting adaptive immunity, primarily engaging the cytotoxic immune response mediated by MHC class I molecules. As a retrovirus, BoDV-1 infiltrates host cells, thereby initiating adaptive immunity and primarily engaging the cytotoxic immune response mediated by MHC class I molecules. The IEDB server remains instrumental for predicting MHC class I molecules (http://tools.iedb.org/mhci/). For peptide prediction, the NetMHCpan EL 4.1, the recommended epitope predictor as of September 2023, was utilized. To enhance prediction accuracy, the NNAlign MA machine learning framework was employed for performance training (30). The results were arranged in descending order according to the predicted scores and presented in a table.

### 2.4 Building vaccine

In order to enhance the safety, reliability, and immunogenicity of the vaccine, epitope screening was conducted based on the criteria of epitope overlap, antigenicity, allergenicity, and toxicity (31). Initially, the predicted B-cell linear epitopes, B-cell nonlinear epitopes, and CTL epitopes were summarized. The overlapping regions between B-cell linear and nonlinear epitopes were then identified. Epitopes that were non-overlapping or too short were excluded. Next, the remaining epitopes were screened for overlap with CTL epitopes. Finally, the overlapping regions of all three types of epitopes were selected for antigenicity detection. These epitopes then underwent antigenicity analysis using the VaxiJen v2.0 tool, applying a selection threshold where the antigenicity score was required to exceed 1. Epitopes that met this criterion were further assessed for allergenicity and toxicity. Toxicity predictions were performed using ToxinPred (https://webs.iiitd.edu.in/raghava/toxinpred), which combines alignment-based methods, motif-based techniques, and machine learning models (32). Only epitopes deemed non-toxic proceeded to the next stage. Allergenicity predictions were carried out with AlgPred (http://crdd.osdd.net/raghava/algpred/submission.htm), which incorporates various predictive methods to improve accuracy in identifying potential allergens (33).

**Figure 2:**
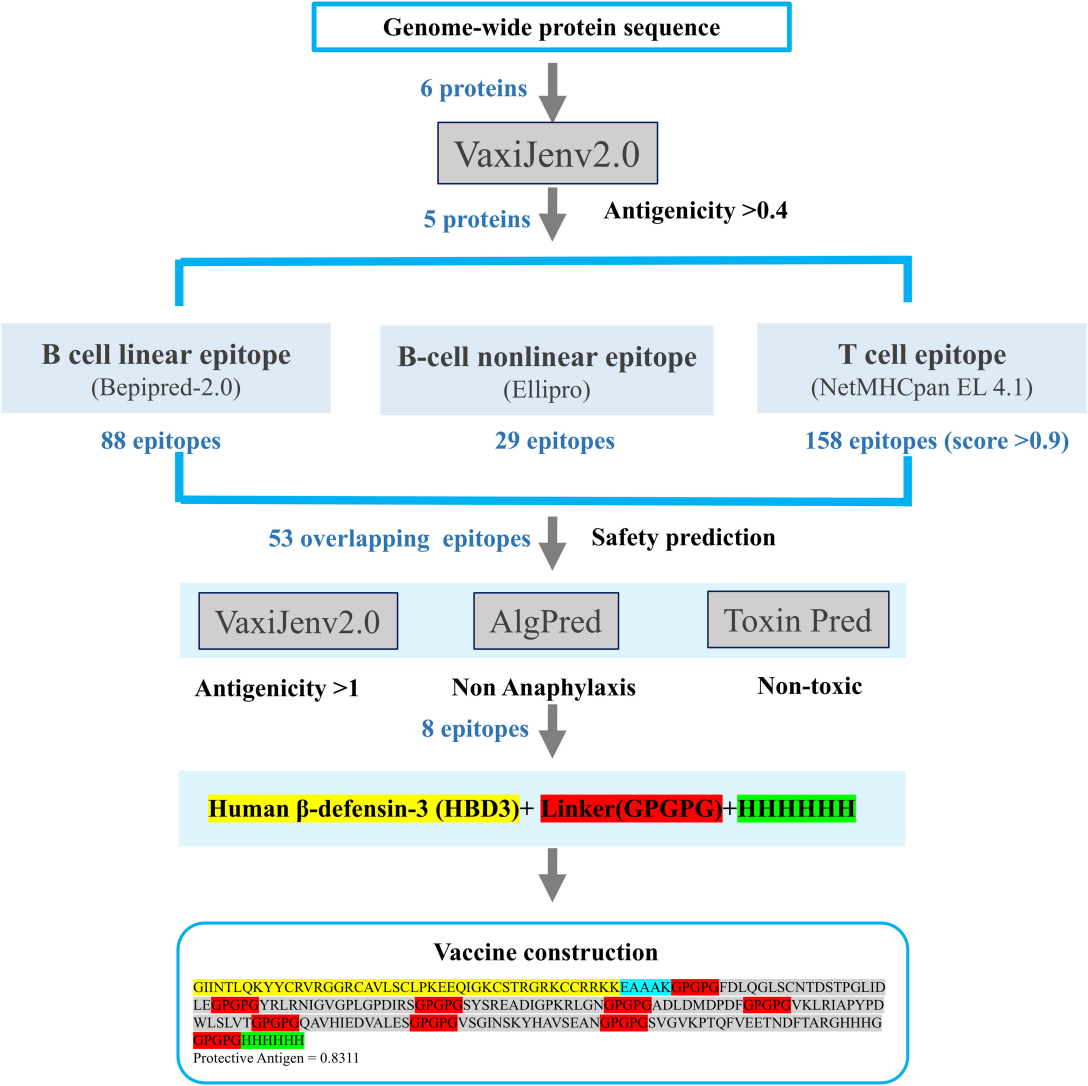
Workflow for epitope selection in vaccine design: Protein sequences were obtained from NCBI, and proteins with antigenicity > 0.4 were selected for epitope construction. Three types of epitopes were predicted using different servers. Overlapping epitopes with antigenicity > 1, non-toxicity, and non-allergenicity were selected, resulting in the construction of three vaccines.

To boost the immune response, the human β-defensin-3 (HBD3) sequence was appended to the N-terminus of the linker sequence using the EAAAK linker. The resulting vaccine fragments were then assembled for further assessment. In order to improve the solubility of the protein expressed by the vaccine and facilitate the purification and advancement of the protein, the Hit-6 labeled sequence was added to the C-terminal. This facilitates binding to metal ions on the chromatographic column, allowing the target protein to attach to the column (34). The above process is shown in **Figure 2** to clearly indicate the process and results of vaccine construction.

### 2.5 Analysis of antigenicity, anaphylaxis, and physicochemical properties

The antigenicity of the final candidate vaccine sequences was evaluated using VaxiJen v2.0, while allergenicity was assessed with AlgPred. The physicochemical properties of the vaccine candidates were analyzed using Expasy ProtParam. The antigenicity evaluation followed the same methodology described in Section 2.1 for protein antigenicity analysis.

For the assessment of allergenic potential, the AlgPred tool (http://www.imtech.res.in/raghava/algpred/) will be utilized. Employing a hybrid approach (SVMc + IgE epitope + ARPs BLAST + MAST) allows for a comprehensive evaluation of allergenicity (35).

Expasy ProtParam (https://web.expasy.org/protparam/) was utilized to analyze the molecular properties of the vaccine sequences, encompassing molecular weight, length, isoelectric point, half-life, and hydropathicity GRAVY values (36). These properties play pivotal roles in determining the stability, solubility, and immunogenicity of the vaccine. Such analysis aids in assessing the rationality of the vaccine design.

To confirm safety against autoimmunity, candidate vaccines underwent screening using the Blastp online server (https://blast.ncbi.nlm.nih.gov/Blast.cgi) for homology screening with human proteins. This step was taken to ensure minimal similarity to human proteins, thereby minimizing autoimmune risks (37).

### 2.6 Evaluation and optimization of secondary and tertiary institutions

The evaluation of secondary structural elements, such as α-helices, β-sheets, and random coils, offers essential information about protein folding and assists in identifying functional domains. As a result, accurately predicting secondary structure is vital for understanding protein function and enhancing vaccine design strategies (38). The amino acid sequence for the designed vaccine was submitted to the PSIPRED server (http://bioinf.cs.ucl.ac.uk/psipred) to predict its secondary structure. This platform provided a comprehensive set of protein prediction and annotation tools, focusing mainly on the structural analysis of proteins and boasting nearly two decades of operational reliability (39). The PSIPRED 4.0, in particular, is highly specialized in secondary structure prediction, with demonstrated accuracy levels, achieving an average Q3 score in the range of 76.5 to 78.3 percent (40). Due to its proven accuracy, it was selected for the secondary structure analysis of the vaccine candidate.

The trRosetta server (https://yanglab.qd.sdu.edu.cn/trRosetta/) was used to predict the tertiary structure of the designed vaccine. It employed a supervised, deformable protein language model with high accuracy (41). To improve the protein structure quality, the GalaxyRefine (http://galaxy.seoklab.org/cgi-bin/submit.cgi?type=REFINE) was employed for structural refinement. The server’s performance was evaluated through CASP10, where it demonstrated superior effectiveness in improving the quality of protein tertiary local structures, thus facilitating structure optimization (42). Protein structure predictions became increasingly accurate and comparable to experimental laboratory results (43).

The Ramachandran plot was created using the SWISS-MODEL workspace (https://swissmodel.expasy.org/assess) to evaluate the stereochemical quality of the final vaccine structure (44). The Ramachandran plot, a core tool in protein structural analysis, visualized the dihedral angles of amino acid backbones within protein models, helping identify errors or deviations in these models. By highlighting allowed and disallowed regions, the plot provided an important quality control mechanism based on known protein structures (45).

Structure validation was also performed using the ProSA-web tool (https://prosa.services.came.sbg.ac.at/prosa.php). ProSA is a widely used program for evaluating potential errors in protein 3D structures and model quality (46). The z-score generated by ProSA indicated the overall quality of the model by assessing the deviation of the total energy of the structure relative to the energy distribution of experimental protein structures (47). A negative z-score typically suggested a stable protein structure with energy levels that align well with native-like conformations.

### 2.7 Immune simulation

To characterize the immune response elicited by the vaccine and evaluate its effectiveness, immunological simulations were conducted using the C-ImmSim online server (https://kraken.iac.rm.cnr.it/C-IMMSIM/). C-ImmSim, which implemented the Celada-Seiden model, is a versatile tool for exploring multiple aspects of immune responses and predicting vaccine efficacy (48). Recorded parameters included T-cell and B-cell responses, as well as the detection of immunoreactive substances, to provide a detailed assessment of the immune responses induced by the vaccine. The simulation was configured to run for 100 steps across three vaccine administrations, with injections at steps 1, 32, and 64 while other simulation settings were kept constant. To assess the impact of varying adjuvant concentrations on the immune response, adjuvant concentrations were adjusted to 100, 1000, and 10,000 to observe changes in the predicted immune response outcomes.

As a validation step for simulation reliability, the classical hepatitis B virus vaccine was employed as a reference. A computational simulation of this reference vaccine was performed to evaluate whether the immune response predictions aligned with previously reported experimental results, ensuring the reliability of our approach.

### 2.8 Molecular docking

The immune response is enhanced through successful interactions between vaccines and immune cell receptors. In this study, two servers were utilized to conduct docking simulations between the vaccine constructs and specific immune cell receptors, namely toll-like receptor-4 (TLR4, 3FXI) and major histocompatibility complex I (MHCI, 4WUU). To validate the binding affinity between the designed vaccine structure and the target receptors, docking analysis was performed using the ClusPro server. ClusPro is an online docking server that employs a fast Fourier transform (FFT) algorithm and has excellent performance in rigid protein-protein docking (49).

The HDOCK server was employed to predict the binding complexes of the vaccine candidates and immune molecules. This tool applied the ff02 force field and the OBC1 model of MM/GBSA to refine predictions of protein-protein binding free energy for accuracy in binding affinity assessment (50). The server generates ten high-scoring predictions along with relevant binding data. After review, the most suitable results were selected for subsequent analysis.

### 2.9 Molecular dynamics simulations

To examine the binding stability and interaction details between vaccine and receptor molecules, molecular dynamics simulations were employed to assess the binding dynamics of the ligand-receptor complex. The WeMol website (https://wemol.wecomput.com/) was ultimately used to perform simulations and conduct further analyses. The molecular dynamics simulation procedures on WeMol were powered by Gromacs software (51). The simulation workflow included preparing the PDB structure, protonating proteins, parameterizing protein properties, solvating, conducting molecular dynamics simulations, and generating and analyzing trajectories. This method translated Gromacs’s intricate coding into a user-friendly format with numerical data and output files, thus simplifying the simulation workflow.

The optimized vaccine protein-receptor docking complex (including TLR4 and MHC-I) was uploaded in the PDB file to initiate molecular dynamics (MD) simulation. Preparation for the simulation began by protonating the protein, setting the pH value to 7, and selecting the AMBER03 force field along with the SPC water model to complete the parameterization of the GMX receptor. The molecular system was subsequently placed in a water box for solvation. After configuring these settings, the energy minimization parameters were set with a generation time of 0.01 ps (picoseconds) and a minimum convergence value of 100. This step minimized the system’s energy to eliminate unfavorable atomic overlaps and high-energy configurations. Following energy minimization, the system was equilibrated under constant temperature (NVT) and constant pressure (NPT) conditions, set at 300K and one atmosphere, respectively. Once these parameters were established, molecular dynamics simulation was initiated, with a simulation duration set to 100 ns, as longer simulation times typically yield more accurate results (52). The detailed process provided by the website was illustrated in **Supplementary Figure S1**.

The path file obtained from the simulation was further analyzed. The molecular simulation trajectory was subsequently visualized using Root Mean Square Deviation (RMSD) analysis, and Root Mean Square Fluctuation (RMSF) was used to measure structural flexibility. RMSD is a measure used to quantify the mean deviation of atomic positions from a reference structure over time (53). RMSF quantifies the average deviation of each atom or residue from its mean position during the simulation (54). Visualization and data plotting were conducted with QtGrace to illustrate the results clearly. The stability and structural variation of the complex were assessed by calculating RMSD and RMSF values (55).

Additionally, molecular dynamics simulations were performed to test the vaccine’s structural integrity under different environmental conditions, investigating if the predicted protein antigen exhibits any conformational or activity alterations at varied pH and temperature. The vaccine structure was optimized and analyzed in two environments: the standard laboratory conditions (pH = 7 and 300 K) and a simulated physiological environment (pH = 7.35 and 310 K). Both conditions underwent 100-ns simulations to observe changes in protein structure and function.

### 2.10 In silico cloning and prediction of RNA secondary structure

For the cloning process, the designed vaccine sequence underwent codon optimization for prokaryotic expression systems using Optipyzer (https://www.optipyzer.com/). The Escherichia coli strain K12, a widely used host in protein expression studies, was selected to enhance the expression efficiency of the vaccine protein (56). To ensure effective translation of vaccine genes, rho-dependent transcription termination sites, prokaryotic ribosome binding sites, and restriction endonuclease cutting sites were selected to be avoided (57). Subsequently, the mRNA secondary structure was predicted using the UNAFold Web Server (http://rna.tbi.univie.ac.at/cgi-bin/RNAWebSuite/RNAfold.cgi) (58). It utilizes a dynamic programming algorithm to predict the minimum free energy (MFE) of a single RNA or DNA sequence presented in plain text or FASTA format. The output includes a mountain map that displays the predicted secondary structure along with the average base pair probabilities, providing a visual representation of the structural dynamics of the sequence. To ensure optimal protein solubility, the ExPASy Translate Tool was used to convert the optimized DNA sequence into an amino acid sequence, followed by solubility assessment using the Scratch Protein Predictor (59). SnapGene 6.0.2 software (http://www.snapgene.com) was utilized to introduce XhoI (158) and SacI (190) restriction endonuclease sites at the N-terminal and C-terminal regions of the vaccine. Finally, the vaccine sequence was inserted into the pET-28(+) plasmid to obtain the final expression vector.

## 3 Results

### 3.1 Protein selection

All proteins encoded by the entire BoDV-1 genome were retrievable from the NCBI database by downloading the NCBI RefSeq assembly (GCF_002366305.1), BoDV-1 protein sequences. The ID number of each protein and its corresponding FASTA format file were recorded. **Table 1** provided the NCBI accession numbers and antigenicity evaluation results for six specific proteins: the X protein, nucleoprotein, phosphoprotein, matrix protein, glycoprotein, and RNA-dependent RNA polymerase.

**Table 1:** NCBI sequence numbers and antigenicity evaluation results of 6 proteins of BoDV-1 whole genome.

Accession Number	Protein name	Antigenic value of protein
YP_009272535.1	X protein	0.5833
NP_042020.1	nucleoprotein	0.4727
NP_042021.1	phosphoprotein	0.3664
NP_042022.1	matrix protein	0.6696
NP_042023.1	glycoprotein	0.4695
NP_042024.3	RNA-dependent RNA polymerase	0.4918

The accession number is the NCBI reference sequence number. The threshold for the VaxiJen score is set at 0.4.

To identify antigenic protein sequences, each protein sequence in the genome was individually evaluated for antigenicity. In this study, the antigenicity of six target proteins was evaluated using the VaxiJen server. The phosphoprotein (NP_042021.1) had an antigenicity score of 0.3664, which fell below the predefined threshold of 0.4; therefore, it was excluded from further analysis. Specifically, the X protein exhibited an antigenicity value of 0.5833, the nucleoprotein 0.4727, the matrix protein 0.6696, the glycoprotein 0.4695, and the RNA-dependent RNA polymerase 0.4918. These proteins were thus selected as primary candidates for antigenic peptide prediction.

### 3.2 Prediction of the B-cell epitopes

**Figure 3:**
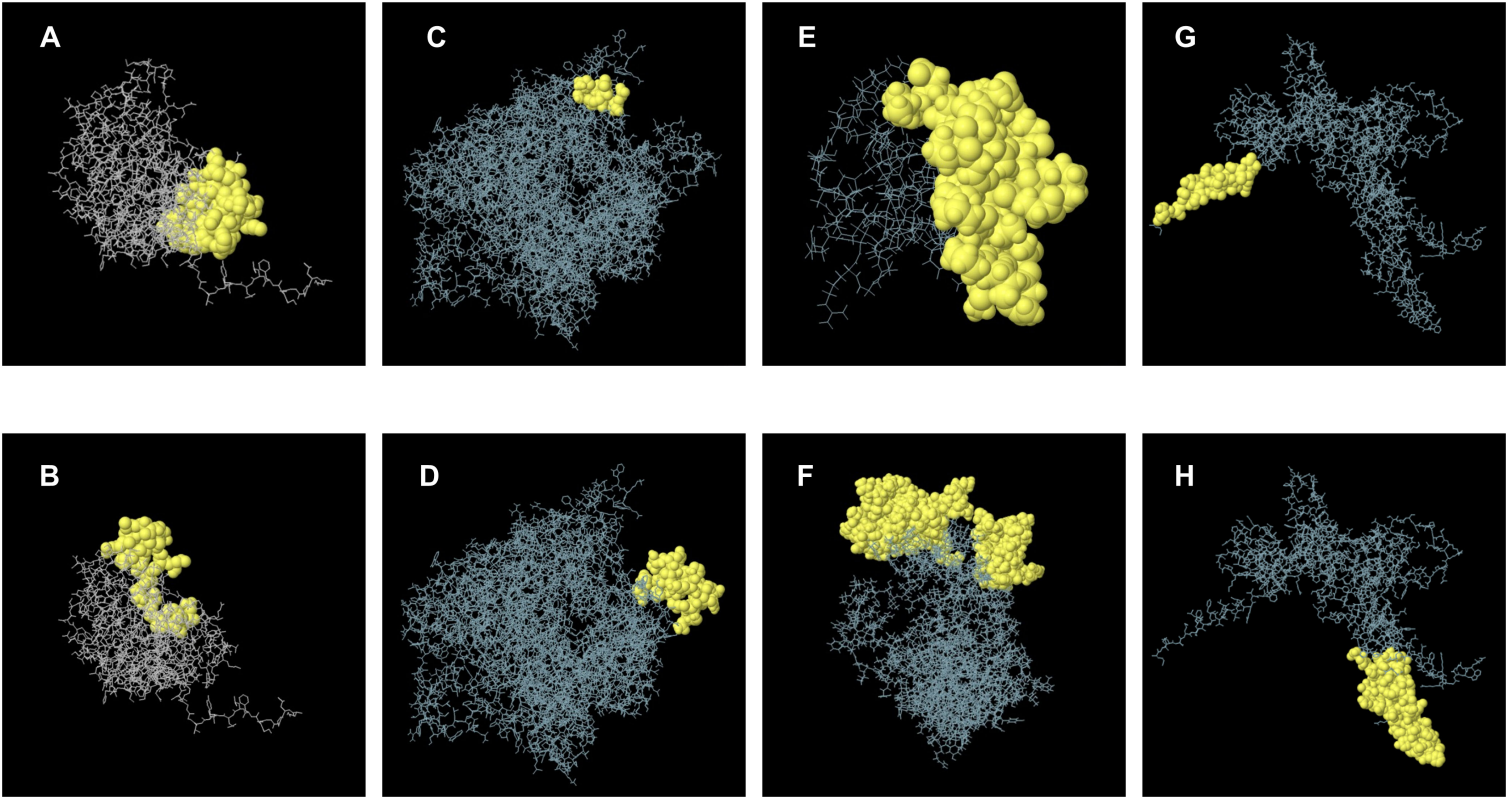
A schematic diagram illustrating discontinuous B-cell epitopes mapping within the 3D structure. **(A, B)** represent B-cell discontinuous epitopes predicted from the nucleoprotein. **(C, D)** represent discontinuous epitopes predicted by RNA-dependent RNA polymerase. (E) represents a discontinuous epitope corresponding to the X protein. **(F)** represents a discontinuous epitope of a matrix protein. **(G, H)** represents B cell structural epitopes predicted by glycoprotein.

We employed the BepiPred 2.0 to predict linear epitopes based on B lymphocyte affinity. The resulting data were downloaded and evaluated for overlap with the previously identified B-cell epitopes, a total of 88 epitopes. The results indicated that the X protein had 1 epitope, the nucleoprotein had 11 epitopes, the matrix protein had 6 epitopes, the glycoprotein had 15 epitopes, and the RNA-dependent RNA polymerase included 55 epitopes. Detailed epitope information was recorded in **Supplementary Table S2**. For B-cell nonlinear epitope prediction, ElliPro software was utilized. However, the three-dimensional structures of the X protein, RNA-dependent RNA polymerase, and glycoprotein were not available. Therefore, trRosetta was used for structure prediction, and its 3D structure was visualized using Structure Assessment (**Supplementary Figure S2**). Following optimization on GalaxyRefine, the prediction with the highest GDT-HA score was selected for further nonlinear epitope prediction (**Supplementary Table S1**). Some of the predicted structural epitopes were shown in **Figure 3**. B-cell epitopes were critical antigenic targets within humoral immunity in the adaptive immune response. All predicted epitopes across the five proteins were recorded to enable overlapping screening with T-cell epitopes in subsequent steps.

### 3.3 Prediction of the epitopes of T-Cell

The identification of T lymphocyte epitopes for the retrovirus BoDV-1 was indeed a crucial aspect of the adaptive immune response. Cytotoxic T lymphocytes (CTLs) played a vital role in defending against viral diseases, making the prediction of CTL epitopes particularly important.

To identify these epitopes, the protein sequences were analyzed using the NetMHCpan 4.1 server. T-cell epitopes with high antigenicity scores and overlapping with B-cell epitopes were selected for integration, totaling 53 overlapping epitopes. The results indicated that the X protein had 1 epitope, the nucleoprotein had 9 epitopes, the matrix protein had 3 epitopes, the glycoprotein had 10 epitopes, and the RNA-dependent RNA polymerase included 30 epitopes. All of these epitopes were used for antigenicity prediction with VaxiJen so that high-antigenicity epitopes could be subsequently selected for final vaccine construction. Epitope information and prediction sequences were shown in **Supplementary Table S3**.

### 3.4 Construction of multi-epitope vaccine

Based on their overlap and antigenicity, epitopes with an antigenicity score greater than 1 were ultimately chosen for vaccine construction. In total, one epitope from the glycoprotein (NP_042031.1), one epitope from the matrix protein (NP_042022.1), and six epitopes from the RNA-dependent RNA polymerase (NP_042024.3) were selected for subsequent vaccine construction. One overlapping epitope of the X protein and nine overlapping epitopes of the nucleoprotein were excluded due to their antigenicity being below 1. The entire epitope prediction and screening process described above is shown in **Figure 2**. The eight selected epitopes were rigorously evaluated for anaphylaxis and toxicity. None exhibited allergic or toxic effects, indicating that the epitope-based vaccine was likely safe and non-toxic. These eight epitopes, along with their respective details, were presented in **Table 2**.

**Table 2:** Overlapping epitopes with the antigenicity >1, non-allergic, non-toxic.

Start	End	Peptide	Length	Protective Antigen		Anaphylaxis	Toxicity
NP_042023.1 glycoprotein							
23	42	FDLQGLSCNTDSTPGLIDLE	20	1.3052		NON	Non-Toxin
NP_042022.1 matrix protein							
123	139	YRLRNIGVGPLGPDIRS	17	2.0186		NON	Non-Toxin
NP_042024.3 RNA-dependent RNA polymerase							
36	50	SYSREADIGPKRLGN	15	1.6946		NON	Non-Toxin
287	295	ADLDMDPDF	9	1.6857		NON	Non-Toxin
743	758	VKLRIAPYPDWLSLVT	16	1.4869		NON	Non-Toxin
852	863	QAVHIEDVALES	12	1.3262		NON	Non-Toxin
1291	1305	VSGINSKYHAVSEAN	15	1.0398		NON	Non-Toxin
1315	1338	SVGVKPTQFVEETNDFTARGHHHG	24	1.0205		NON	Non-Toxin

The accession number is the NCBI reference sequence number. Based on this fact, the subsequent vaccine construction will indeed incorporate overlapping epitopes with antigenicity scores greater than 1.

To enhance the immunogenicity of the vaccine, the sequence of human β-defensin-3 was appended to the N-terminus and connected to the cellular epitope via the linker EAAAK. The epitopes were linked using GPGPG connectors, culminating in the final vaccine design. The Hit-6 tag was attached to the C-terminus via a GPGPG linker, followed by a HHHHHH tag. Similarly, the final constructed vaccine sequence is shown in **Supplementary Figure S3**.

### 3.5 Analysis of antigenicity, anaphylaxis, and physicochemical properties

The antigenicity value of the constructed vaccines was 0.8311. Based on the results, the vaccine was selected for subsequent natural testing. A hybrid method (SVMc + IgE epitope + ARPs BLAST + MAST) via the AlgPred server was utilized to assess vaccine sensitization. Prediction by the SVM method, based on amino acid composition, showed a threshold of -0.4. The predicted coverage reached 89.45%, with a positive predictive value of 18.21% and a negative predictive value of 71.24%, indicating the vaccine’s non-allergenic nature.

The ProtParam tool was utilized to assess the physicochemical properties of the vaccine structure, with the aim of evaluating its stability. The evaluated subunit vaccine comprised 3287 atoms, with a chemical formula of C_1032_H_1617_N_317_O_313_S_8_. Notably, the vaccine contained 22 negatively charged residues and 25 positively charged residues. The vaccine construct consisted of 218 amino acids, with a molecular weight of 23.729 kDa and a predicted theoretical pI value of 8.48. The calculated instability index of the vaccine was 30.57, indicating that the protein is stable. The aliphatic index of the vaccine was 63.01, demonstrating high thermal stability. The GRAVY of the vaccine structure was -0.614, indicating that the protein was hydrophobic. All results were presented in **Supplementary Table S4**.

Furthermore, Blastp analysis revealed no significant homology with human proteins, suggesting a low risk of autoimmune reactions (**Supplementary Figure S4**). In conclusion, the findings from the antigenicity, anaphylaxis, and physicochemical assessments indicated that the vaccine exhibited high antigenicity, stability, safety, and reliability.

### 3.6 Evaluation and optimization of secondary and tertiary institutions

To ensure vaccine safety, a homology analysis was performed, and a comparative homology analysis was performed by aligning the vaccine’s protein sequence with that of human counterparts, indicating the minimal risk of triggering an autoimmune response. The predicted secondary structure indicated that the vaccine comprises 6.6% α-helix, 16.6% β-sheets, and 77.4% coils (**Supplementary Figure S5**).

**Figure 4:**
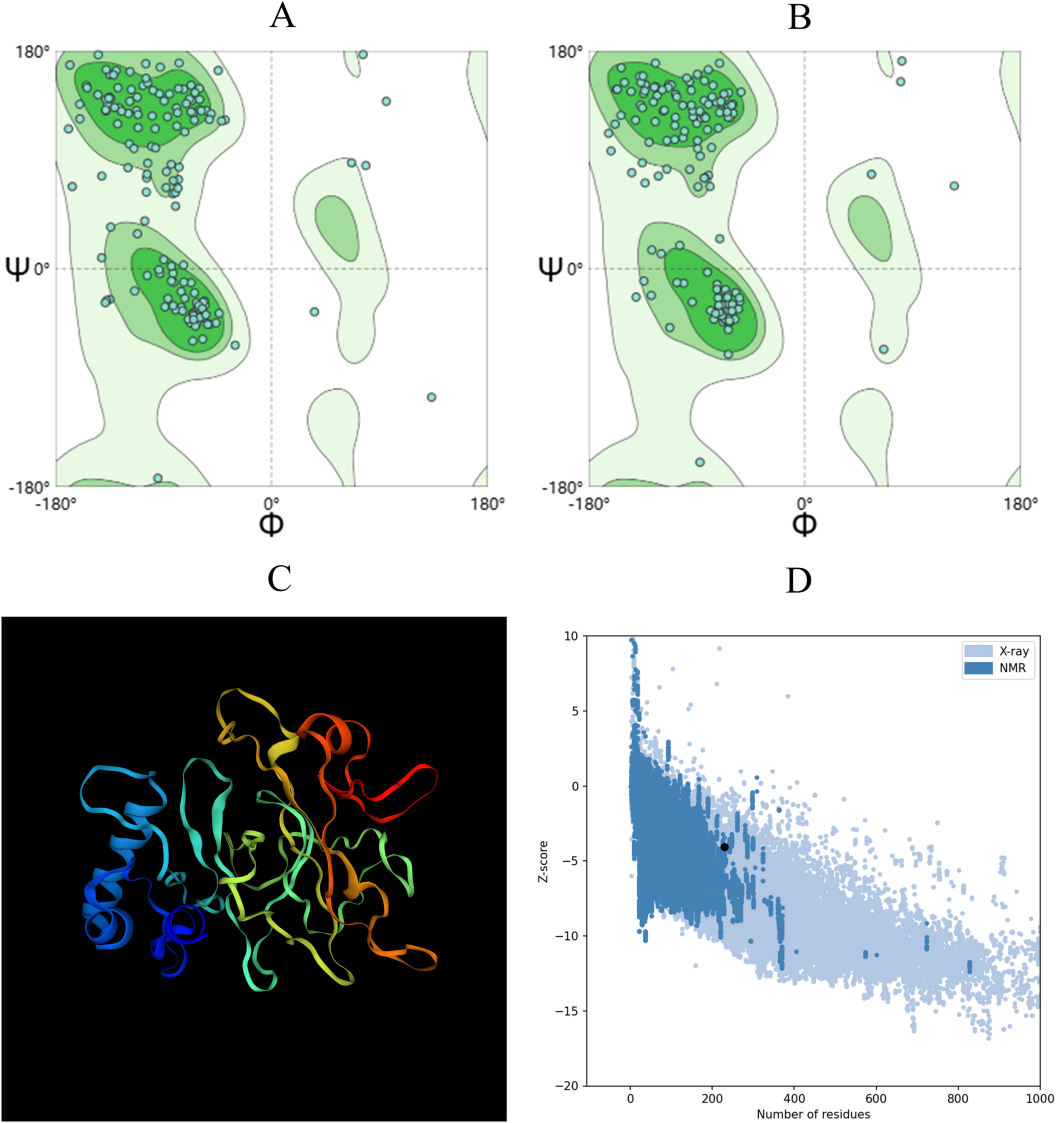
The 3D structure of the vaccine was evaluated using the Ramachandran plot analysis in SWISS-MODEL. **(A)** Ramachandran plot depicting the initial model. **(B)** Ramachandran diagram illustrating the enhanced model. **(C)** The diagram showcases the optimized vaccine structure. **(D)** The Z-score of the optimized structure is provided below.

The tertiary structure of the vaccine sequence was initially modeled using trRobetta and then further refined and analyzed on the Galaxy platform. Five models were generated on the GalaxyRefine server using an algorithm. Detailed information about these models was provided in **Supplementary Table S5**. Model 2 achieved the highest score with a GDT-HA (Global Distance Test - High Accuracy) value of 0.9727, making it the final selected structure (**Figure 4C**).

A superior vaccine model should have a greater proportion of residues in the Ramachandran favored region and fewer in the outlier and rotamer regions. Both the initial model from trRobetta and the optimized model from GalaxyRefine were evaluated using the SWISS-MODEL workspace, as shown in **Figures 4A, B**. The initial model demonstrated that 88.99% of residues were in the Ramachandran favored region, 2.64% were in the Ramachandran outlier region, and 0% were in the rotamer outlier region. The refined model exhibited a higher occupancy in the Ramachandran favored region, with 92.07% of residues, while the Ramachandran outlier region contained 0.88%, and the rotamer outlier region contained 0.57%.

The quality and potential errors of the final 3D vaccine model were further assessed using ProSA-web. A lower Z-score indicated higher model quality and fewer potential errors. The refined model achieved a Z-score of -3.94 (**Figure 4D**), indicating a significant improvement in model quality.

### 3.7 Immune simulation

**Figure 5:**
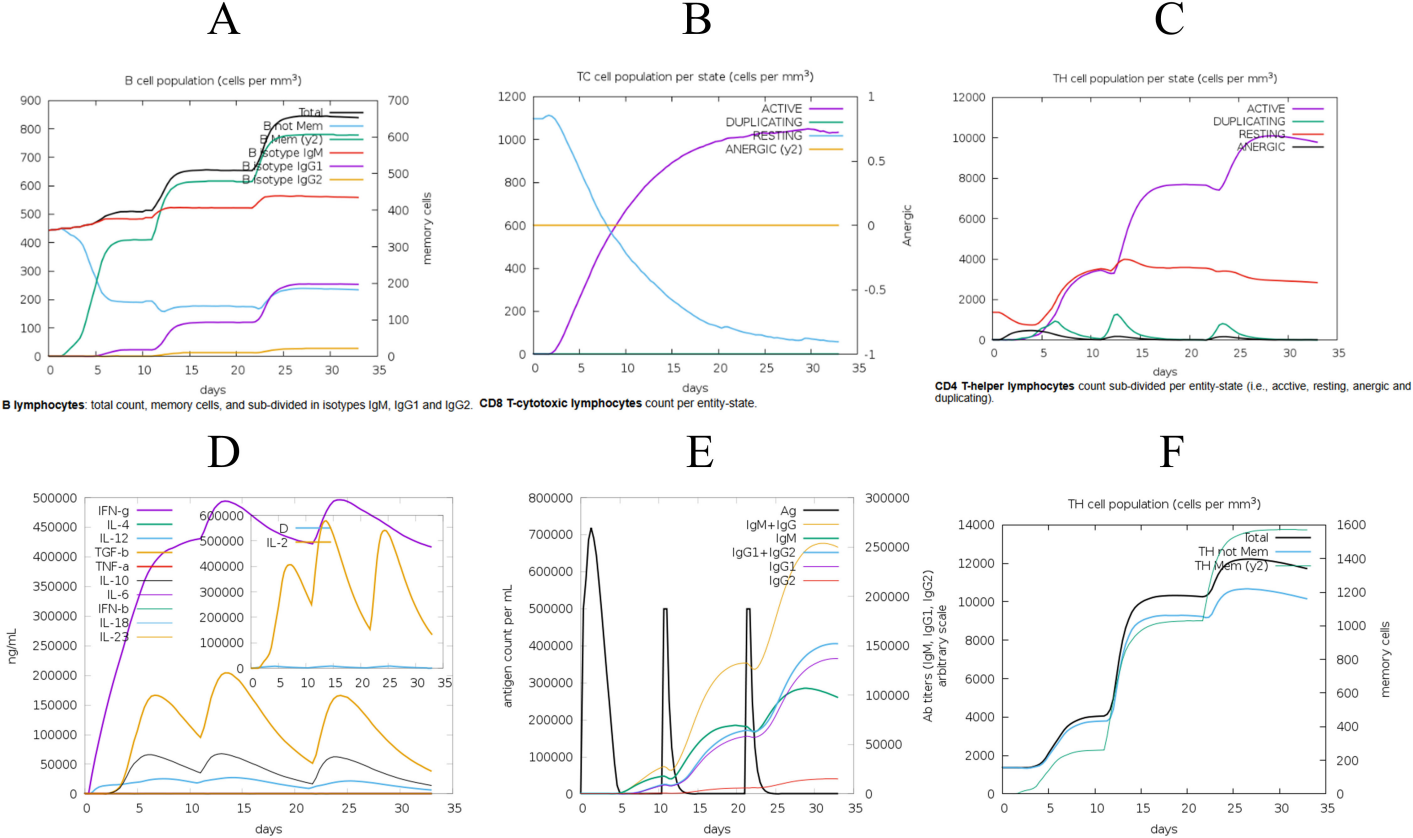
The C-ImmSim server was employed to evaluate the induced immune response. **(A)** Post-vaccination assessment of B-cell response. **(B)** Analysis of cytotoxic T-cell immune response following vaccination. **(C)** Examination of helper T-cell immune response. **(D)** Concentrations of cytokines and interleukins measured post-vaccination. **(E)** Levels of specific antigens and immunoglobulins following vaccination. **(F)** Quantification of T helper (Th) cells and memory cell populations after immunization.

The designed vaccine was simulated using the C-ImmSim online server to assess its immune response. The simulation results demonstrated enhanced immune activation, with a significant increase in memory B-cell titers (**Figure 5A**), higher levels of cytotoxic and helper T-cells (**Figures 5B, C**), and increased IFN-γ levels during exposure (**Figure 5D**). The secondary and tertiary immune responses showed increased levels of antibodies (IgG1, IgG2, IgM) and a rapid decrease in antigen concentrations, indicating the activation of humoral immunity and robust immune cell proliferation *in vivo* (**Figure 5E**). Administering three doses of the vaccine resulted in a marked increase in specific T-cells, especially memory T-cells, suggesting the successful induction of a long-lasting immune memory (**Figure 5F**). These results indicated that the vaccine candidate effectively stimulates host cells to produce a robust immune response. The immune simulation results of the classical hepatitis B vaccine closely aligned with the responses observed in real vaccine immunizations, such as significant increases in IL-2- and TNF-α-producing cells, particularly CD8+ T cells (60, 61). as shown in **Supplementary Figure S6**.

Simultaneously, the immune simulation results showed significant improvements after adjusting the adjuvant dose, particularly in the levels of immune molecules such as IgM and IgG, which increased with higher doses, as illustrated in **Supplementary Figure S7**. This indicates that modifications to the adjuvant can indeed influence the outcome of immune simulations.

### 3.8 Molecular docking

**Figure 6:**
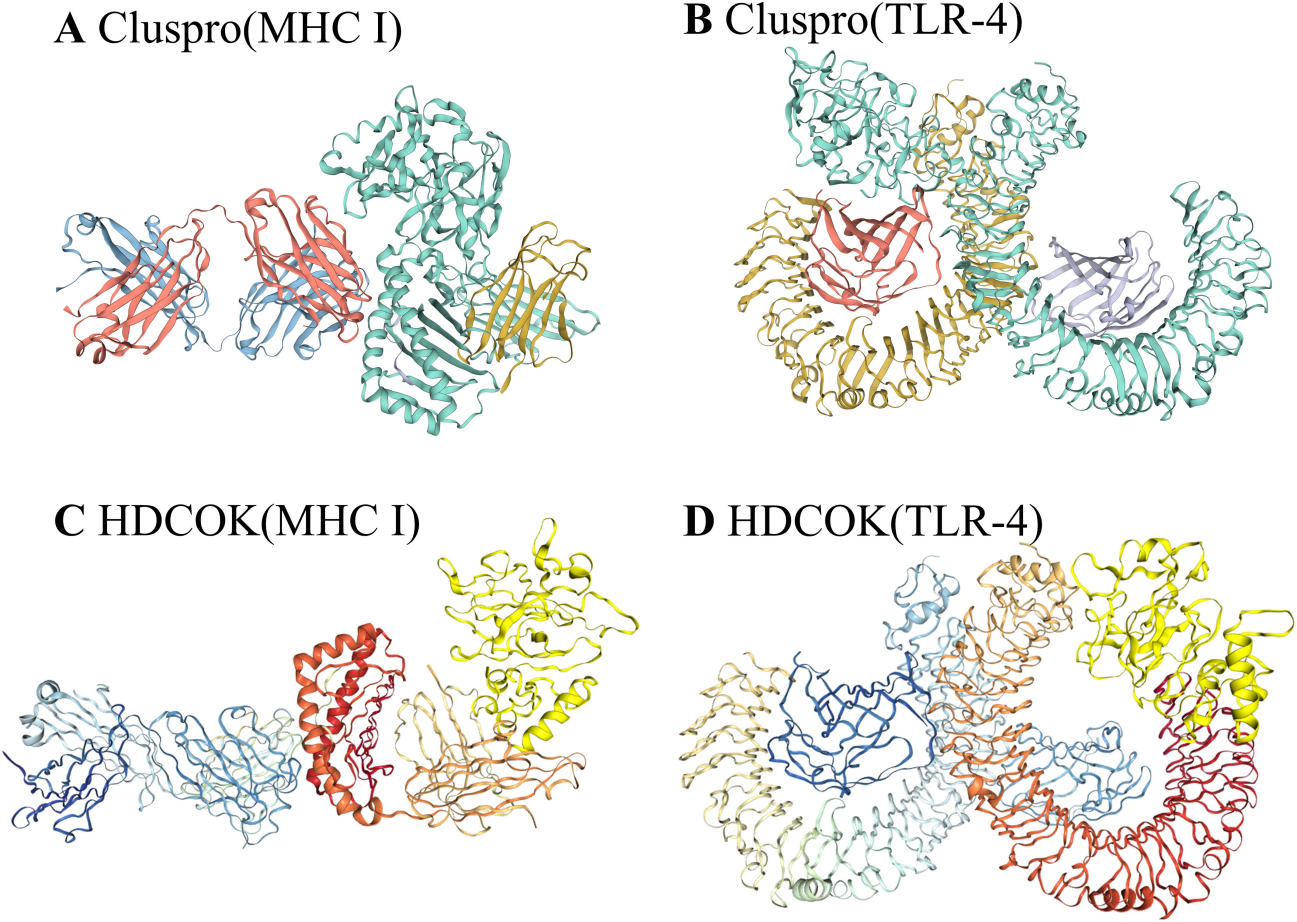
Results of vaccine docking with MHC I and TLR4. **(A)** Predicted vaccine-MHC I binding in Cluspro. **(B)** Predicted vaccine-TLR4 binding in Cluspro. **(C)** Predicted vaccine-MHC I binding in HDCOK with modifications. **(D)** Predicted vaccine-TLR4 binding in HDCOK with modifications.

To evaluate the binding affinity of the vaccine constructs with antigen receptors (TLR4, MHC-I), protein-protein molecular docking was conducted using the ClusPro 2.0 docking server. Human MHC-I and TLR4 were used as receptors, while the 3D model of the vaccine served as the ligand. The binding energy scores indicated the strength of the vaccine-receptor interactions, with lower scores reflecting stronger binding. A total of 29 complexes were generated between the vaccine and MHC-I, and the complex with the lowest binding energy score of -810.7 kcal/mol was selected as the most favorable interaction (**Figure 6A**). Similarly, 29 docking complexes between the vaccine and TLR4 were identified. Among these, the complex with the lowest binding energy score of -912 kcal/mol was considered the most favorable interaction (**Figure 6C**).

For further validation, HDOCK was employed to generate additional docking models and relative score tables. Research indicated that models with more negative docking scores were indicative of higher receptor affinity. The models were ranked by their docking scores, with model 1 being the most favorable among the top 10 predicted models. Specifically, the docking score for the MHC-I-vaccine protein complex was -263.06 kcal/mol, with a confidence score of 0.9056 and a ligand root mean square deviation (RMSD) of 33.42 Å (**Table 3**; **Figure 6B**). The TLR4-vaccine complex, on the other hand, exhibited a docking score of -287.33 kcal/mol, with a confidence score of 0.9397 and a ligand RMSD of 43.77 Å (**Table 3**; **Figure 6D**).

**Table 3:** The score of the optimal model for molecular docking.

Receptor	ClusPro		HDOCK		
	Center	Lowest energy	Docking Score	Confidence Score	Ligand rmsd (Å)
MHC-I	-810.7	-810.7	-263.06	0.9056	33.42
TLR4	-724.5	-912	-287.33	0.9397	43.77

### 3.9 Molecular dynamic simulation

**Figure 7:**
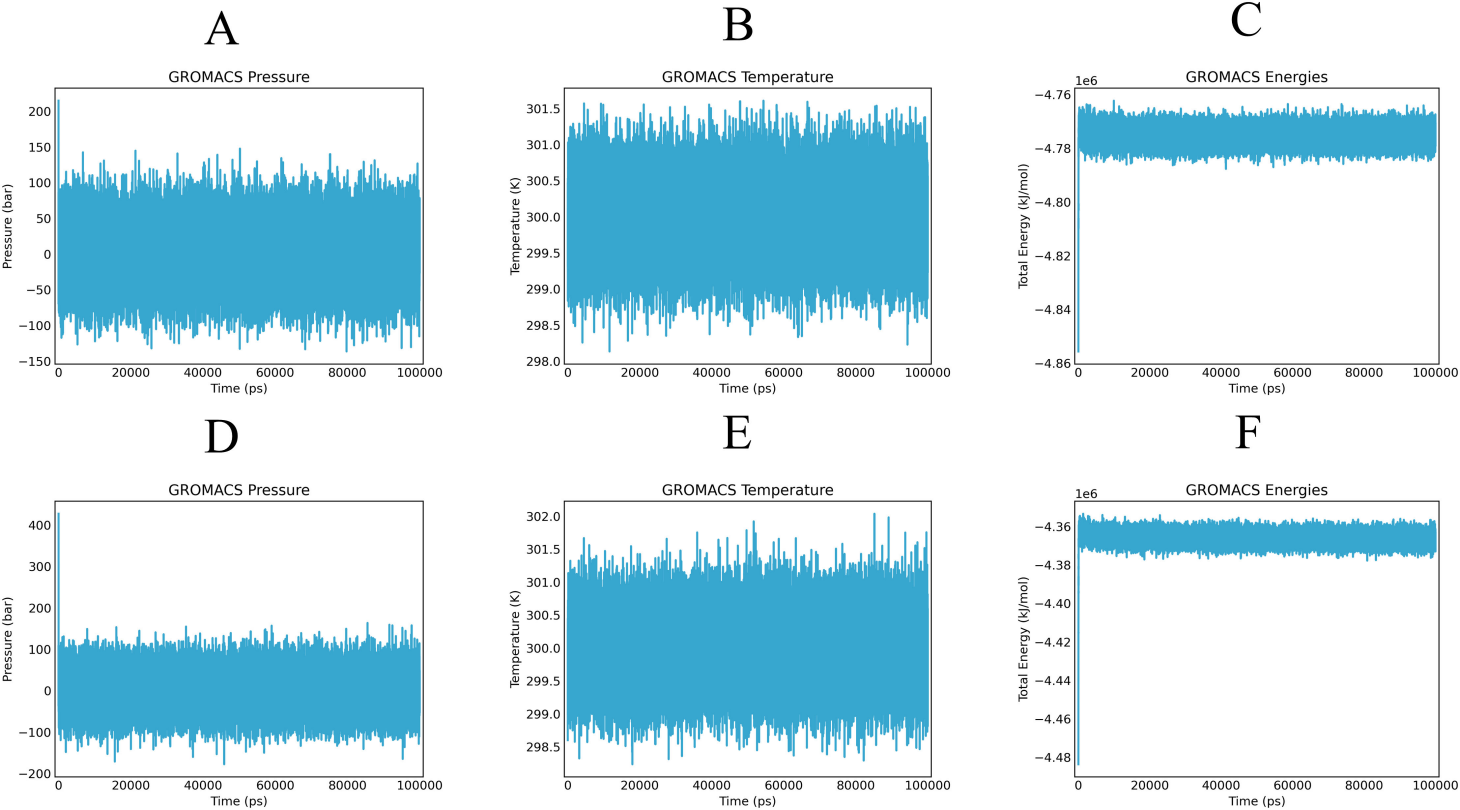
Alterations in pressure, temperature, and energy of vaccines and immune receptors are noted during molecular dynamics simulation. **(A, D)** illustrate the pressure values of the vaccine-MHC-I and TLR4, respectively, which display fluctuations around the 1 bar threshold. **(B, E)** demonstrate the overall temperature during the equilibrium phase (maintained at a constant 300 K) of the vaccine-MHC-I and TLR4 complexes. **(C, F)** present the fluctuations in interaction energy over the course of the simulation period of the vaccine-MHC-I and TLR4 complexes.

By simulating the docking complex, the stability of the vaccine-receptor complex was evaluated using the Gromacs-based platform, WeMol. Several analyses were conducted, including energy minimization, pressure, and temperature assessments, as well as potential energy calculations. In the simulation, the temperature and pressure of the vaccine-MHC-I and TLR4 complexes were maintained at approximately 300 K and 1 atm, respectively (**Figure 7**), indicating stable system operation. During the simulation, the interaction energy of MHC-I varied within the range of -4.77×10^6^ kJ/mol (**Figure 7C**), while the interaction energy of TLR4 fluctuated around -4.36×10^6^ kJ/mol (**Figure 7F**). These results demonstrate that both MHC-I and TLR4 exhibited strong binding affinity with the vaccine, showing good stability and interaction characteristics.

**Figure 8:**
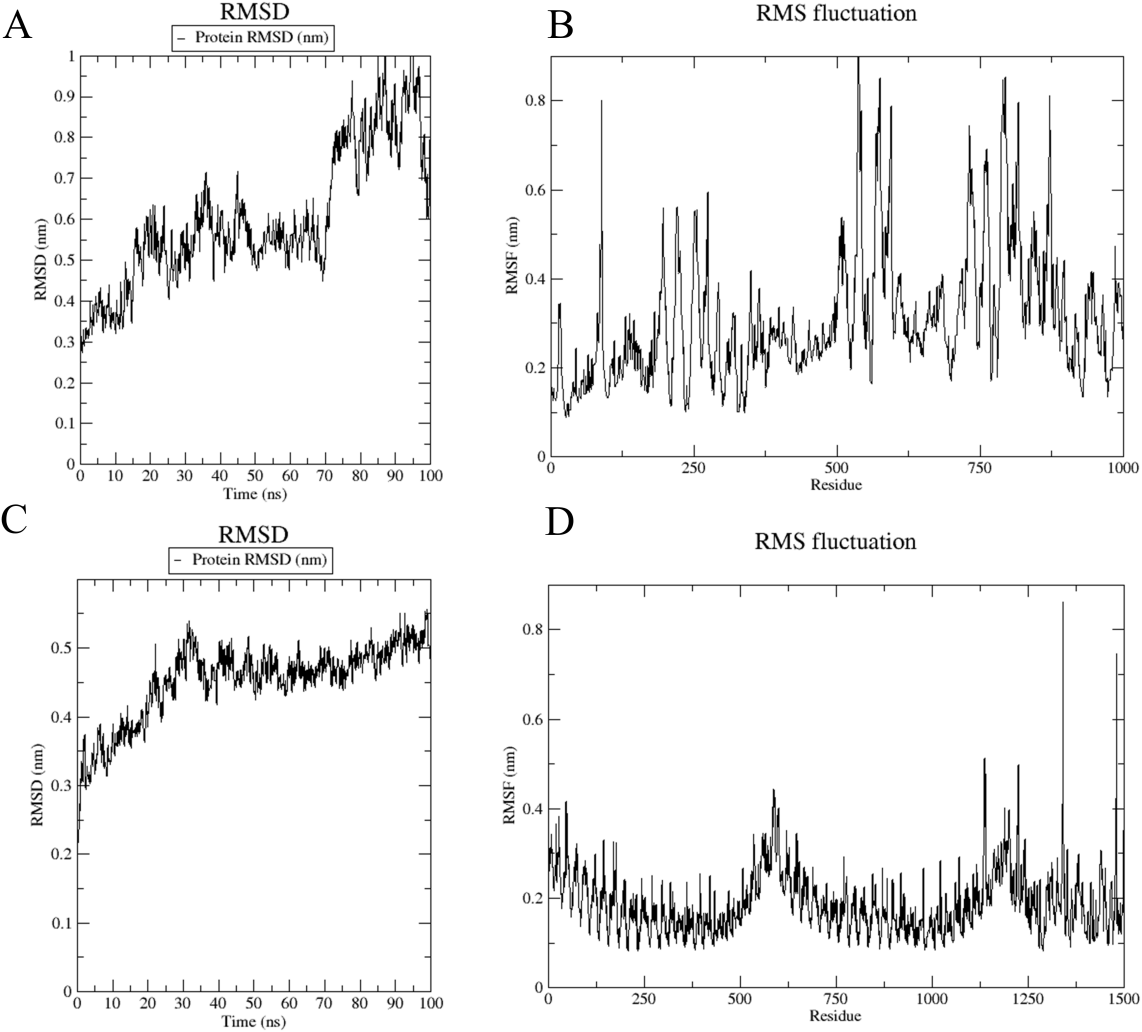
Root mean square deviation (RMSD) chart and root mean square fluctuation (RMSF) chart. **(A, B)** The RMSD and RMSF plots were generated from molecular docking simulations of the vaccine with the MHC-I complex. **(C, D)** The RMSD and RMSF plots were generated from molecular docking simulations of the vaccine with the TLR4 complex.

The root-mean-square deviation (RMSD) analysis illustrates structural fluctuations in the vaccine-immune receptor complex. For the vaccine-MHC I complex, RMSD exhibited a rapid initial increase, stabilizing between 0.4-0.6 nm from 0-70 ns (**Figure 8A**), followed by an increase to 0.8 nm with greater fluctuation amplitude post-70 ns. Root-mean-square fluctuations (RMSF) measure the degree to which each atom or residue of a complex fluctuates relative to its average position. The analysis revealed higher overall values for residues 0-1000, with significant peaks at residues 500 and 875, indicating increased structural flexibility (**Figure 8B**).

In the vaccine-TLR4 complex, RMSD showed an initial rise to 0.5 nm within the first 30 ns, followed by relative stability at 0.6 nm (**Figure 8C**). The RMSF values for residues 0-1250 were low, indicating reduced flexibility, while residues 1250-1500 exhibited higher RMSF values, suggesting increased flexibility in these regions (**Figure 8D**).

After comparing the RMSD and RMSF in both standard and simulated *in vivo* environments, the results showed minimal significant changes. However, RMSD exhibited greater stability at 310 K, where it initially rose gradually from 0 to 0.8 nm in the standard environment. In contrast, in the 310 K environment, RMSD experienced a sudden increase to 0.8 nm before stabilizing around 0.7 nm with minimal fluctuations thereafter. The results are shown in **Supplementary Figure S8**. This indicated that while the overall trends remained consistent, the elevated temperature may induce more abrupt changes in the dynamics of the system.

### 3.10 In silico cloning and prediction of RNA secondary structure

**Figure 9:**
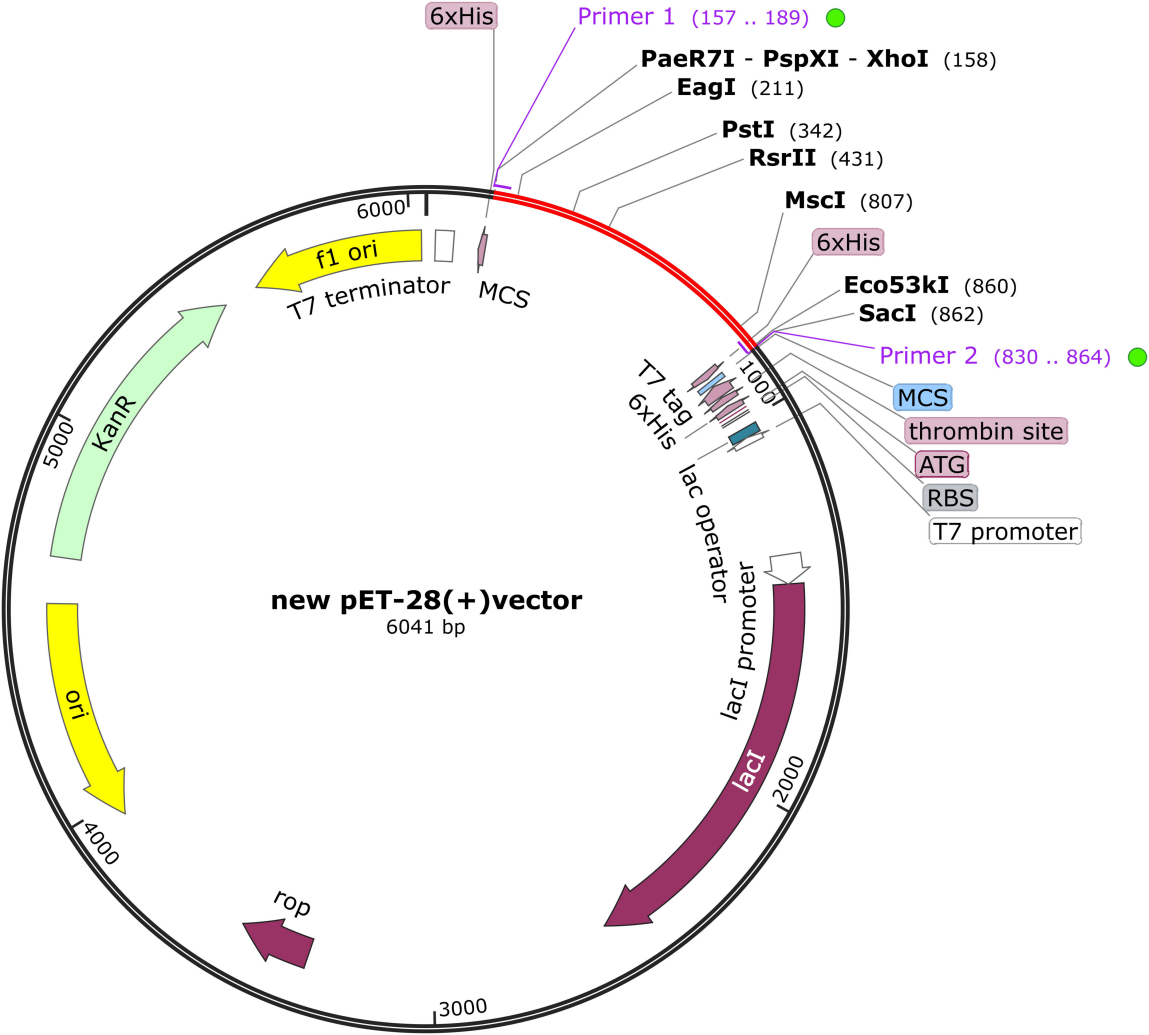
The BoDV-1 vaccine was subjected to *in silico* cloning within the pET28a (+) vector. The red areas highlight the vaccine, while the black areas denote the expression vector, pET-28a (+).

For the cloning process, the Optipyzer tool was used to optimize the codon usage, as shown in **Supplementary Figure S8**. This optimization led to significant improvements in the sequence characteristics, with the GC content increasing from 58.12% to 60.29%, and the Codon Adaptation Index (CAI) rising from 0.69 to 0.88, thereby enhancing the potential for efficient gene expression in host organisms. Detailed sequence information is provided in **Supplementary Figures S9A, B**. A CAI value close to 1 indicated that the optimized codon usage was well-adapted to the host organism’s preferred codon usage (62). Additionally, the optimized sequence was transcribed and translated into an amino acid sequence, as shown in **Supplementary Figure S9C**, and the measured solubility was 0.947237. The optimized codon sequence consists of 690 nucleotides. Restriction sites XhoI and SacI were incorporated at the N- and C-termini, respectively, of the developed vaccine’s nucleotide sequence. This sequence was then inserted into the pET28a (+) vector between the XhoI and SacI sites, as illustrated in **Figure 9**, resulting in a construct of 6041 base pairs. The RNAfold program predicted the RNA secondary structure with a minimum free energy of -314.40 kcal/mol, indicating stability; a more negative value reflects a more favorable configuration (**Supplementary Figure S10A**). The ridge map shows higher values between positions 200-500, indicating a higher density of base pairs in this region, which may suggest the potential for forming a relatively stable secondary structure (**Supplementary Figure S10B**).

## 4 Discussion

BoDV-1 is an emerging zoonotic disease, clinically characterized by its potential to cause a relatively rare, yet highly fatal, form of encephalitis (63, 64). Due to the absence of long-term surveillance, limitations exist in understanding the infectivity and prevalence of the virus (65, 66). The virus’s capacity to infect a diverse range of hosts poses a major threat to human society if it becomes a pandemic (67). However, no standard treatment guidelines currently exist for BoDV-1 infection, and the development of vaccines has progressed slowly (68, 69). Therefore, there is an urgent need for specific treatment or preventive measures against the virus. So far, no research has successfully developed a vaccine targeting the virus to prevent BoDV-1 infection in humans, nor has any such vaccine advanced to the clinical stage. In a 1998 study, Oliver Planz and Lothar Stitz used multiple vaccinia virus recombinants expressing a single BoDV-1 specific protein and confirmed the presence of T-cell epitopes on the viral protein p40. However, the study did not thoroughly explore the immune responses triggered by other BoDV-1 specific proteins, which limited its applicability in virus prevention (70). Additionally, the 2018 poultry Borna virus disease vector vaccine study by Samer Sadeq Hameed and Susan Payne focused on poultry and failed to achieve effective results in preventing viral infection (71). Immunoinformatics offers a promising approach for identifying and designing novel vaccine candidates against BoDV-1 (72). Our study employed immunoinformatics methods to design a preventive vaccine against BoDV-1, providing a blank for the development of human BoDV-1 infection vaccine.

In this study, we formulated a genome-wide protein vaccine against BoDV-1. Epitope prediction was conducted from 5 proteins with positive antigenicity. Vaccines crafted to target the entire genome of a pathogen offer broader protection against the targeted pathogen (73). The development of vaccines targeting whole-genome proteins can elicit a broader spectrum of humoral and cellular immune responses, as they incorporate multiple epitopes (74). Additionally, including a variety of viral proteins helps to minimize the risk of the virus evading immune surveillance through mutation (75). This approach has been explored for a variety of pathogens, including human respiratory syncytial virus and SARS-CoV-2 (76, 77). Finally, T/B cell overlap epitopes were selected for vaccine construction. T-cell and B-cell epitopes play a key role in both humoral and cellular immunity (78). The TELEISSIF short peptide (129 to 137) selected as an MHC I epitope exhibited a high score of 0.930882, consistent with previous findings identifying TELEISSI as the immunodominant CTL epitope of BDV p40 in H-2k mice (79). The matrix protein (M) and glycoprotein (G) within our chosen epitopes serve as constituents of the viral lipid envelope, crucially enhancing the generation of infectious particles and the dissemination of BoDV-1 (80).

These epitopes are linked by GPGPG spacers, and EAAAK junctions fuse the adjuvant β-defense protein to the N-terminus to augment immunogenicity. The inclusion of at least one 5-residue spacer is essential to prevent interlinking among epitopes. GPGPG isolators are the optimal choice to achieve this effect (81). Incorporating human beta-defensins at the onset of the vaccine facilitates cellular uptake of DNA and CpG, subsequently amplifying IFN-α production (82). This cytokine plays a crucial role in attracting and mobilizing immune cells to the vaccination site. The systematic recruitment of immune cells is essential for enhancing the specific immune response elicited by the vaccine, thereby potentially improving its efficacy (83). Moreover, Borna virus infections are particularly prevalent among immunocompromised patients, and HBD3 presents specific advantages in this context (84). Additionally, HBD3 exhibits anti-inflammatory properties that may mitigate the inflammatory storm associated with Borna virus, thereby potentially reducing its high mortality rate (85). To enhance the solubility of the vaccine protein and facilitate subsequent purification, a Hit-6 tag was appended to the C-terminus of the vaccine sequence. Although it was found that HHHHHH labels did not significantly increase the solubility of vaccines compared to other soluble markers, such as maltose binding protein (MBP) (86), the compact nature of the Hit-6 label was shown to reduce the impact on vaccine antigenicity and tertiary structure, thereby enabling more accurate predictions for subsequent vaccine evaluations (87, 88). In contrast, using the MBP tag may hinder follow-up testing due to its length (89). Additionally, incorporating metal ions like nickel or cobalt in the chromatographic purification process post-Hit-6 tag addition significantly enhances purification efficiency, thereby improving vaccine accessibility (90).

The immune simulation conducted using the C-ImmSim server has validated its capacity to induce a robust immune response. Significant proliferation of T cells and IgG1 molecules was observed, aligning with previously documented findings. Research has shown the effectiveness of ELISpot in identifying virus-specific T cells to confirm BoDV-1 infection (91). Additionally, IgG1 was identified as the predominant IgG subclass detected against the BoDV-1 antigen in patient sera, indicating that the vaccine elicits targeted immunity against the virus (92). The simulated results for the hepatitis B vaccine aligned closely with actual outcomes, further validating the reliability of the immune simulation (61). Consequently, future experimental validation of vaccines should prioritize the assessment of immune responses related to IgG production and T cell activation (93). Notably, the immune simulation results exhibited significant variation following changes in the adjuvant dose (94, 95), but also emphasizes the importance of considering non-vaccine components, such as adjuvants, in the design process. However, a limitation of the current immune simulation server, C-IMMSIM, is that it permits only quantitative adjustments to the adjuvant value, lacking the capability to modify the type of adjuvant used.

By utilizing molecular docking techniques involving TLR4 and MHC-I molecules, the interaction pattern of the vaccine with these receptors and its presentation to CD8+ T cells was predicted (96). The interaction between Toll-like receptor 4 (TLR4) and major histocompatibility complex class I (MHC I) is critical in the immune response elicited by vaccines. TLRs, particularly TLR4, are pivotal in recognizing viral proteins, as evidenced by experimental studies demonstrating significant alterations in TLR4 signaling pathways in mice infected with Borna disease virus (BoDV-1) (97, 98). These changes result in the enrichment of MyD88 and interferon regulatory factor 5 (IRF5), subsequently leading to the release of various downstream inflammatory mediators. In the context of BoDV-1 infection, MHC I molecules play a crucial role by recognizing and binding to viral antigenic peptides, which activates CD8+ T cells and initiates cytotoxic T lymphocyte (CTL) responses (99). Consequently, both TLR4 and MHC I are identified as viable targets for vaccine development, warranting docking and dynamic molecular simulations to explore their interactions further. The analyses conducted using ClusPro 2.0 and HDOCK servers have validated the binding affinity of the vaccine to the receptor complex. The results of vaccine receptor affinity predictions varied across different servers (100). However, the different results of ClusPro 2.0 and HDOCK also reflect the affinity of the vaccine to the receptor.

Subsequently, molecular dynamics simulations were performed on the docking complex using the Wemol website to assess the overall structure and residues of the immune receptor complex. MD simulations effectively capture the long-term dynamics of vaccine-receptor interactions (101). In the vaccine-MHC I complex, a mutation observed at 70 ns suggests potential instability, reflected by fluctuating RMSD values linked to structural changes in the vaccine or MHC I (102). In contrast, the vaccine-TLR4 complex demonstrated stable RMSD over 100 ns, corroborating experimental results of a strong interaction (103). RMSF analysis revealed significant peaks at residues 500-875 of MHC I and elevated values between residues 1250-1500 of TLR4, indicating increased flexibility in these regions, which may enhance the adaptive immune response by optimizing vaccine-receptor interactions (104). These findings provide critical insights into the conformational dynamics of vaccine-receptor complexes, informing further vaccine optimization and development (105).

To enhance translation efficiency, the Optipyzer was utilized, and the vaccine sequence was cloned into the expression vector p-ET28a (+) for subsequent animal experiments. It is important to note that BmtI cannot be chosen as an enzyme restriction site, as the vaccine contains this site. Selecting this restriction site would likely hinder the vaccine from transcribing a complete and normal sequence (106, 107). This consideration is crucial for ensuring the proper functionality of the vaccine.

This study demonstrates that a vaccine designed against Borna disease virus (BoDV-1) is a promising candidate, warranting further research and development. The need for a vaccine against this virus is urgent, as it exhibits a high fatality rate, and the epidemiology is still a mystery, and once the epidemic will bring great harm to humans (108). Should an outbreak occur, it could cause significant harm to human populations. Furthermore, there is evidence suggesting that the virus has the potential to mutate and spread, leading to substantial social consequences (109). Therefore, this study provides a solid foundation for the rapid development of antiviral vaccines in the future. Certainly, our study has some limitations, further experimental validation is necessary.

## Data Availability

The original contributions presented in the study are included in the article/**Supplementary Material**. Further inquiries can be directed to the corresponding author/s.
