# Supplementary material for: Developing a universal multi-epitope protein vaccine candidate for enhanced borna virus pandemic preparedness
Source: Front Immunol. 2024 Dec 5;15:1427677. doi: 10.3389/fimmu.2024.1427677 (PMC11655343; doi:10.3389/fimmu.2024.1427677)
Supplement: Supplementary file 1 [file Image1.pdf]

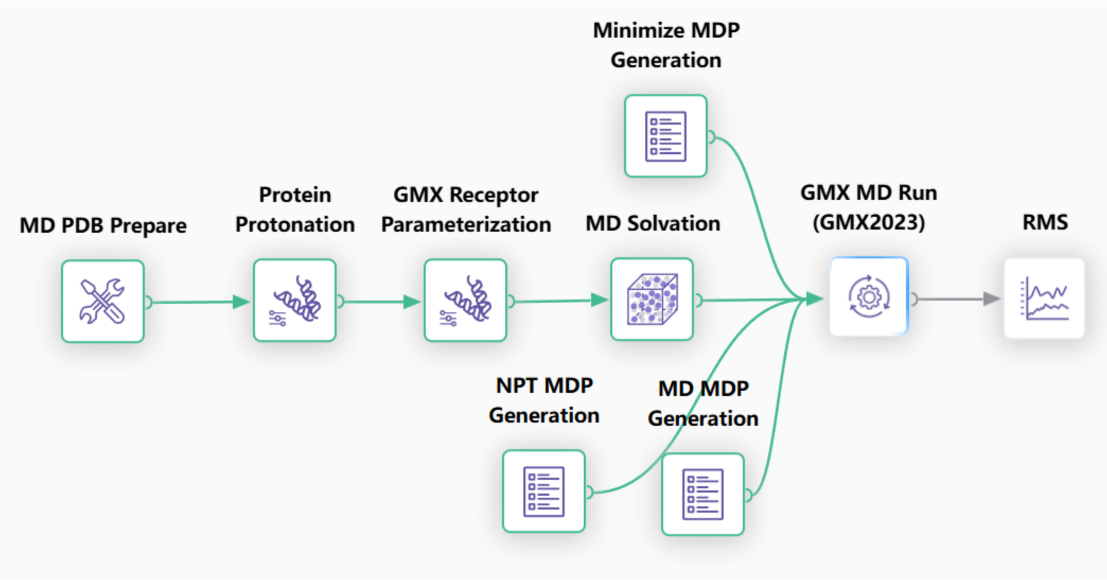

Supplementary Figure 1. Flowchart of the molecular docking module on the wemol website.

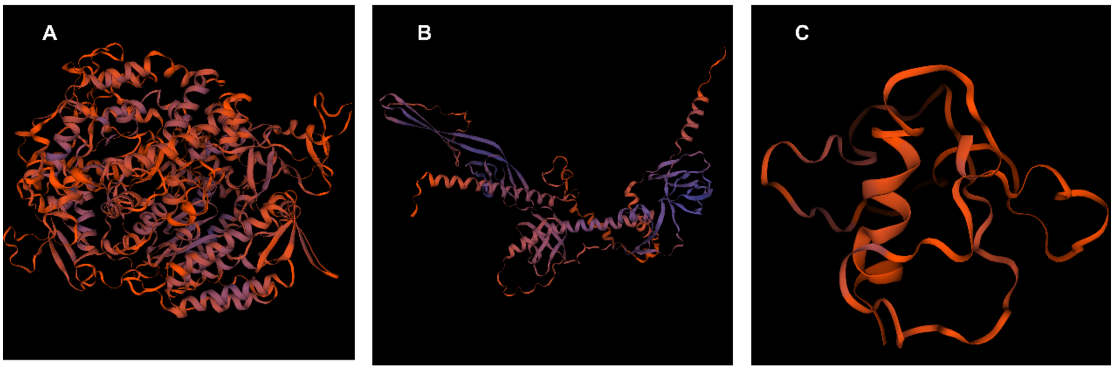

Supplementary Figure 2. SwissModel platform for 3D structure prediction. (A) Prediction model of RNA-dependent RNA polymerase. (B) Glycoprotein prediction models. (C) The prediction model of X protein.

GIINTLQKYYCRVRGGRCAVLSCLPKEEQIGKCSTRGRKCCRRKKEAAAKGP
GPGFDLQGLSCNTDSTPGLIDLEGPGPGYRLRNIGVGPLGPDIRSGPGPGSYSR
EADIGPKRLGNGPGPGADLDMDPDFGPGPGVKLRIAPYPDWLSLVTGPGPGQ
AVHIEDVALESGPGPGVSGINSKYHAVSEANGPGPGSVGVKPTQFVEETNDFT
ARGHHHGGPGPGHHHHHH

Protective Antigen = 0.8311

Supplementary Figure 3. The final vaccine is formed by linking the epitopes with beta-defensin, initially through EAAAK and then through GPGPG.

	Description	Scientific Name	Max Score	Total Score	Query Cover	E value	Per. Ident	Acc. Len	Accession
☑	beta-defensin 3 [Homo sapiens]	Homo sapiens	98.2	98.2	19%	6e-26	100.00%	45	AAV41025.1
☑	beta-defensin-like protein [Homo sapiens]	Homo sapiens	96.3	96.3	19%	8e-25	100.00%	77	ACK99045.1
☑	beta-defensin 103 precursor [Homo sapiens]	Homo sapiens	94.7	94.7	19%	3e-24	100.00%	67	NP_001075020.1
☑	beta-defensin-3 [Homo sapiens]	Homo sapiens	89.7	89.7	19%	2e-22	97.78%	67	AAM62424.1

Supplementary Figure 4. The defensin segment exhibited homology, while the overall vaccine showed no homology with human proteins.

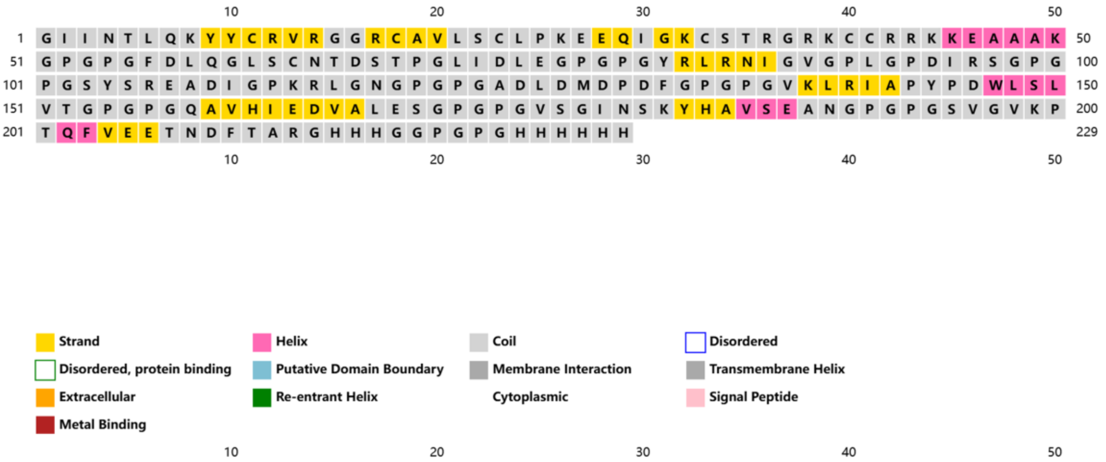

Supplementary Figure 5. The secondary structure of the vaccine was predicted using the PSIPRED server. Beta folds are represented by the color yellow, alpha spirals are represented by the color pink, and random curls are represented by the color gray.

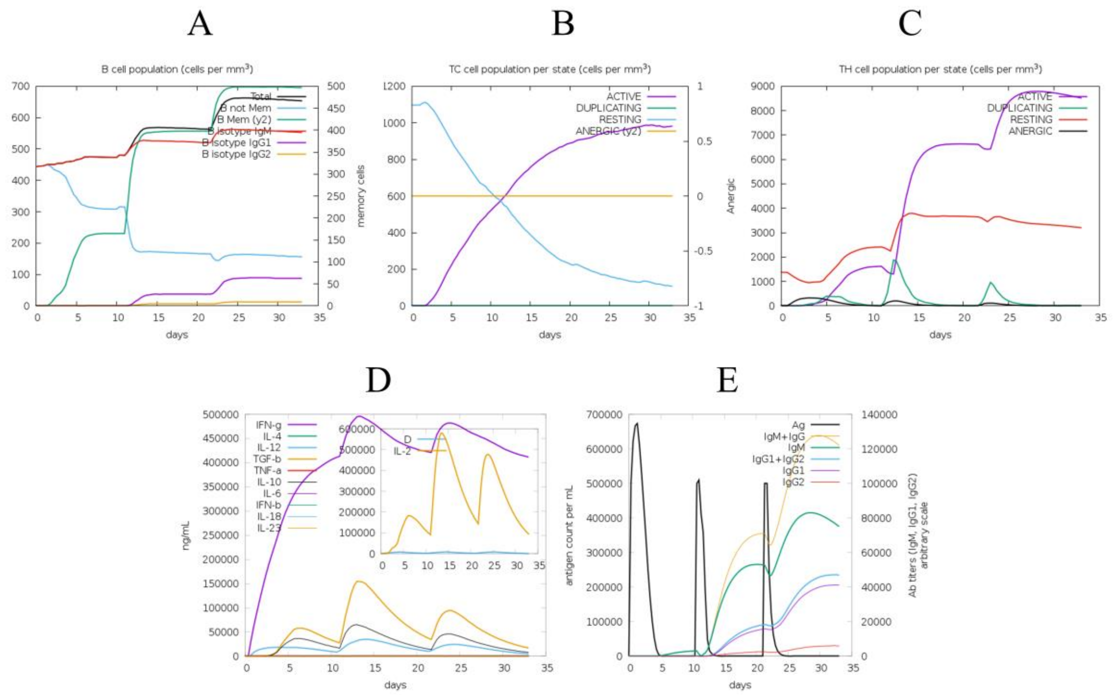

Supplementary Figure 6. Predicted results of hepatitis B vaccine in C-Immsim server. (A) Assessment of b cell response after vaccination. (B) Analysis of cytotoxic t cell immune response after vaccination. (C) Test for helper t cell immune response. (D)

Concentration of cytokines and interleukins. (E) Antigen and immunoglobulins following vaccination.

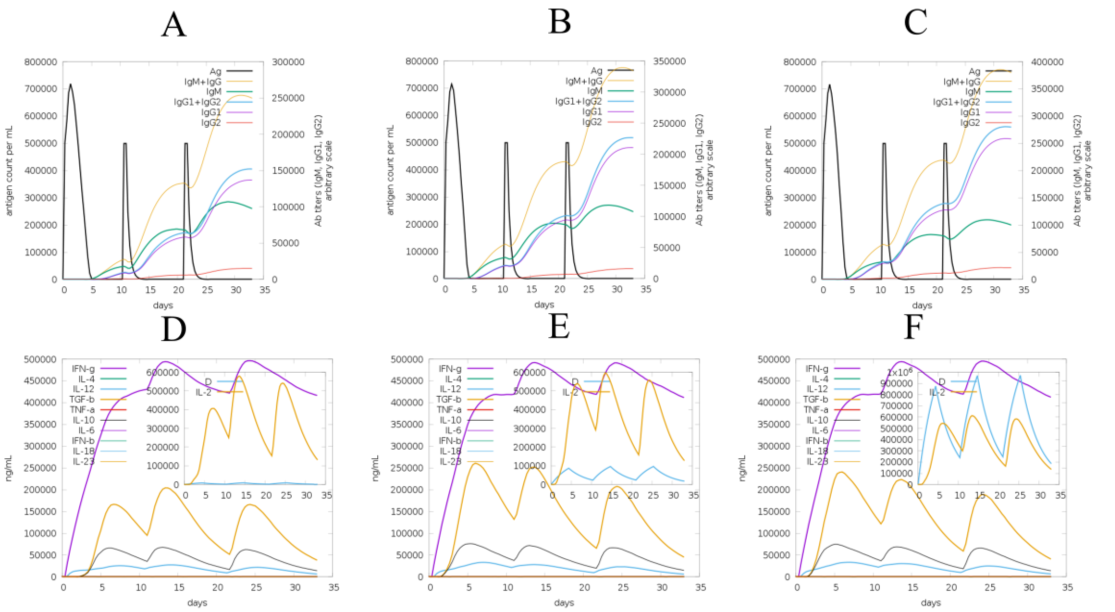

Supplementary Figure 7. (A-C) immunoglobulin proliferation predicted by adjuvant dose 100-1000-1000, respectively. (D-F) adjuvant dose 100-1000-10000, as predicted by cytokine profile induced IFN-γ levels.

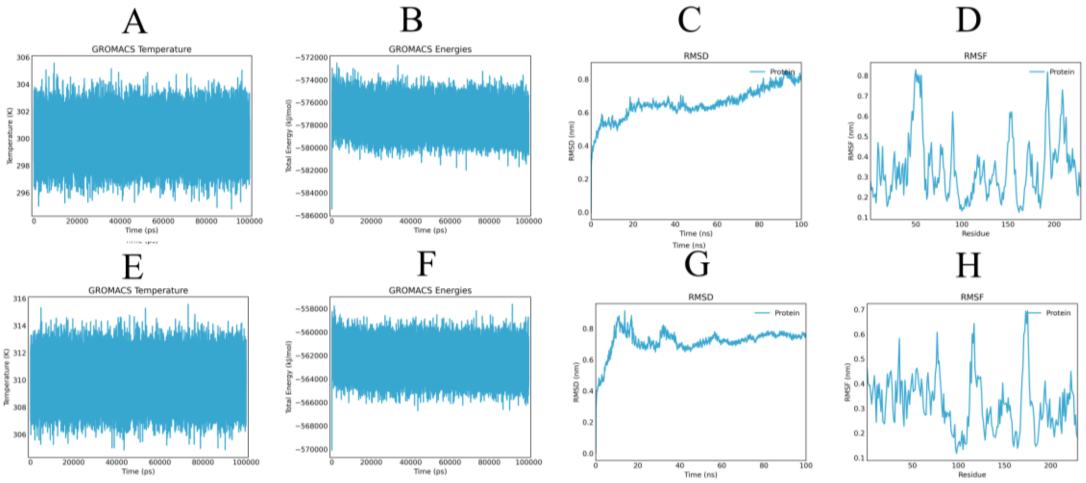

Supplementary Figure 8. A/B represents the temperature and energy parameters set at

300 K and 7.00, while C/D indicates the predicted root mean square deviation (RMSD) and root mean square fluctuation (RMSF). E/F corresponds to the temperature and energy parameters set at 310 K and 7.35, with G/H denoting the predicted RMSD and RMSF values.

A:Unoptimization
GGTATTATCAATACCCTGCAGAAATACTATTGCCGCGTGCGTGGCGGCCGTT
GTGCCGTTCTCTCTTGCCTGCCGAAAGAAGAGCAAATTGGTAAGTGCTCTA
CTCGTGGTCGTAAATGCTGCCGTCGCAAAAAAGAAGCGGCCGCCAAGGGG
CCGGGCCCAGGTTTCGATTTACAGGGCTTATCCTGTAATACCGACTCGACTC
CGGGATTGATCGATCTGGAGGGTCCGGGTCCTGGCTACCGCCTGCGTAACA
TTGGCGTAGGCCCACTGGGTCCGGATATTCGTTCCGGTCCGGGTCCAGGTT
CATATAGCCGTGAAGCGGATATCGGCCCTAAGCGCCTGGGGAATGGGCCGG
GACCTGGTGCCGATCTCGACATGGATCCGGATTTCGGTCCCGGGCCGGGGG
TGAAGTTACGCATTGCCCCGTATCCGGATTGGCTTAGCTTAGTCACCGGTCC
TGGGCCGGGCCAGGCAGTTCACATCGAAGATGTCGCCTTAGAATCTGGGCC
GGGACCGGGTGTGTCTGGGATCAATTCCAAATATCATGCAGTCAGCGAAGC
AAACGGTCCGGGCCCGGGTAGCGTGGGCGTCAAACCAACACAGTTTGTGG
AAGAGACCAACGATTTTACTGCTCGTGGACACCACCACGGCGGACCGGGT
CCGGGCCACCATCATCATCACCATTAA
GC=58.12%, CAI=0.69

B:Optimized sequence
GGCATTATTAATACCTTACAGAAATATTATTGTCGTGTTCGTGGCGGCCGTTG
CGCAGTGCTGAGTTGCCTGCCGAAAGAAGAACAGATTGGTAAATGCAGCA
CCCGTGGCCGTAAATGCTGCCGCCGTAAAAAAGAAGCAGCAGCGAAGGGT
CCGGGCCCGGGCTTCGATCTGCAGGGCCTGAGCTGCAACACCGATAGCAC
CCCGGGCCTGATTGATCTGGAGGGCCCGGGTCCGGGTTACCGTCTGCGCAA
CATTGGCGTCGGTCCGCTGGGTCCGGATATTCGCAGCGGCCCGGGCCCGGG
TAGCTATAGTCGTGAAGCCGATATTGGTCCGAAACGTCTGGGTAATGGTCCG
GGTCCGGGCGCGGATCTGGACATGGATCCGGATTTCGGCCCGGGTCCGGGC
GTAAAACTGCGCATTGCCCCGTACCCAGATTGGCTGAGCCTGGTGACGGGC
CCGGGCCCGGGTCAGGCGGTGCATATCGAAGATGTTGCCCTGGAATCAGGC
CCGGGTCCGGGCGTTAGCGGTATTAACAGCAAATATCACGCGGTGTCGGAG
GCAAATGGTCCGGGCCCGGGCAGTGTCGGCGTGAAACCGACCCAGTTTGT
GGAAGAAACCAATGATTTTACCGCGCGTGGCCATCATCACGGCGGCCCGGG
CCCGGGCCATCATCATCATCATCATTAA
GC=60.29%, CAI=0.88

C: Transcribed translation content:
GIINTLQKYYCRVRGGRCAVLSCLPKEEQIGKCSTRGRKCCRRKKEAAAKGP
GPGFDLQGLSCNTDSTPGLIDLEGPGPGYRLRNIGVGPLGPDIRSGPGPGSYSR
EADIGPKRLGNGPGPGADLDMDPDFGPGPGVKLRIAPYPDWLSLVTGPGPGQ
AVHIEDVALESGPGPGVSGINSKYHAVSEANGPGPGSVGVKPTQFVEETNDFT

ARGHHHGGPGPGHHHHHH

SOLUBLE with probability 0.947237

Supplementary Figure 9. (A) DNA sequence predicted from the amino acid sequence. (B) Optimized DNA sequence following warp code adjustments. (C) Amino acid sequence resulting from the transcription and translation of the optimized DNA sequence.

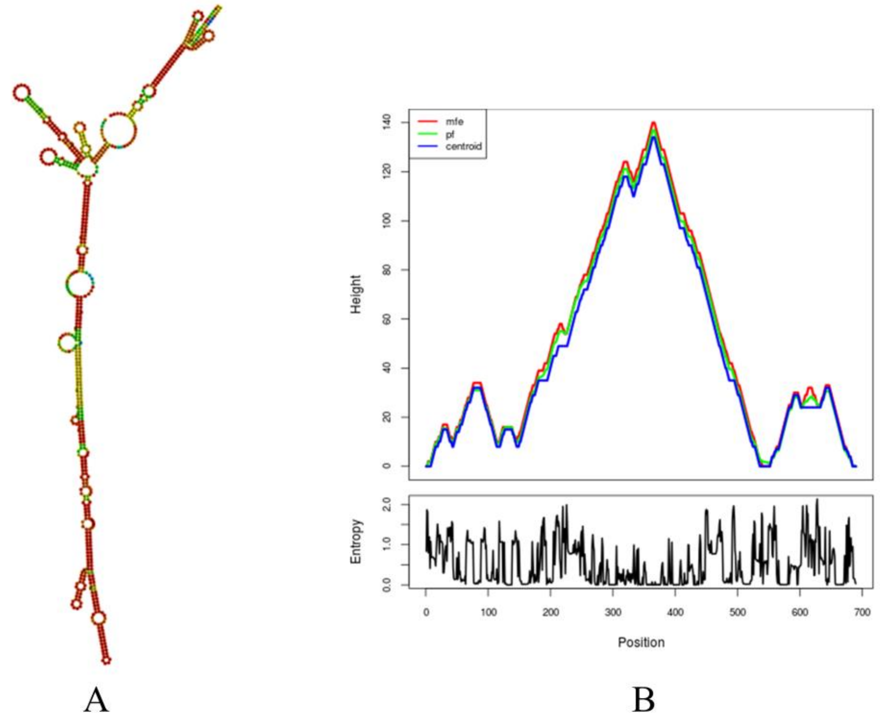

Supplementary Figure 10. Figure (A) shows the RNA secondary structure predicted by the RNAfold program. Figure (B) presents the corresponding forecast ridge map.
