# Supplementary material for: Developing a universal multi-epitope protein vaccine candidate for enhanced borna virus pandemic preparedness
Source: Front Immunol. 2024 Dec 5;15:1427677. doi: 10.3389/fimmu.2024.1427677 (PMC11655343; doi:10.3389/fimmu.2024.1427677)
Supplement: Supplementary file 2 [file Table1.docx]

**Supplementary Table 1.1.** Structural optimization by the Galaxy server glycoprotein.

| Model | GDT-HA | RMSD | MolProbity | Clash score | Poor rotamers | Rama favored |
| --- | --- | --- | --- | --- | --- | --- |
| Initial | 1.0000 | 0.000 | 1.311 | 2.7 | 0.0 | 96.2 |
| MODEL 1 | 0.9672 | 0.373 | 1.541 | 10.5 | 0.9 | 98.0 |
| MODEL 2 | 0.9771 | 0.358 | 1.634 | 9.9 | 0.4 | 97.4 |
| MODEL 3 | 0.9627 | 0.398 | 1.596 | 9.8 | 0.4 | 97.6 |
| MODEL 4 | 0.9573 | 0.390 | 1.687 | 9.5 | 1.1 | 97.2 |
| MODEL 5 | 0.9707 | 0.379 | 1.648 | 10.3 | 0.4 | 97.4 |

**Supplementary Table 1.2.** Optimization results of RNA-dependent RNA polymerase.

| Model | GDT-HA | RMSD | MolProbity | Clash score | Poor rotamers | Rama favored |
| --- | --- | --- | --- | --- | --- | --- |
| Initial | 1.0000 | 0.000 | 2.549 | 20.4 | 1.8 | 89.6 |
| MODEL 1 | 0.9700 | 0.353 | 2.081 | 19.9 | 1.1 | 96.1 |
| MODEL 2 | 0.9706 | 0.358 | 2.087 | 18.7 | 0.8 | 95.4 |
| MODEL 3 | 0.9710 | 0.367 | 2.076 | 18.5 | 0.9 | 95.5 |
| MODEL 4 | 0.9706 | 0.355 | 2.141 | 19.4 | 1.2 | 95.6 |
| MODEL 5 | 0.9655 | 0.378 | 2.160 | 19.4 | 1.3 | 95.7 |

**Supplementary Table 1.3.** Optimization results of X protein.

| Model | GDT-HA | RMSD | MolProbity | Clash score | Poor rotamers | Rama favored |
| --- | --- | --- | --- | --- | --- | --- |
| Initial | 1.0000 | 0.000 | 4.042 | 31.4 | 24.7 | 43.5 |
| MODEL 1 | 0.8592 | 0.680 | 2.823 | 32.7 | 0.0 | 71.8 |
| MODEL 2 | 0.8563 | 0.682 | 3.105 | 30.6 | 2.7 | 74.1 |
| MODEL 3 | 0.8506 | 0.675 | 2.878 | 29.8 | 1.4 | 72.9 |
| MODEL 4 | 0.8563 | 0.674 | 3.133 | 32.7 | 2.7 | 74.1 |
| MODEL 5 | 0.8563 | 0.687 | 3.117 | 29.8 | 2.7 | 71.8 |

**Supplementary Table 2.** It shows the prediction results of B-cell epitopes.

| No. | Start | End | Peptide | Length |
| --- | --- | --- | --- | --- |
| **NP_042023.1 glycoprotein** | | | | |
| 1 | 23 | 42 | FDLQGLSCNTDSTPGLIDLE | 20 |
| 2 | 48 | 49 | HT | 2 |
| 3 | 52 | 52 | E | 1 |
| 4 | 82 | 82 | K | 1 |
| 5 | 86 | 103 | GFFGSYSADRIINRYTGT | 18 |
| 6 | 106 | 116 | GCLNNSAPEDP | 11 |
| 7 | 158 | 176 | CSTVSQQELESGKAMLSDG | 19 |
| 8 | 178 | 194 | TLTYTPYILQSEVVNKT | 17 |
| 9 | 206 | 227 | KIVSFDEFRRSYSLTNGSYQSS | 22 |
| 10 | 238 | 256 | SSCRPRLKRRRRDTQQIEY | 19 |
| 11 | 259 | 259 | H | 1 |
| 12 | 262 | 270 | RPTLKDAWE | 9 |
| 13 | 329 | 361 | ESTYYPPVDYNGRKYFLNDEGRLQTNTPEARPG | 33 |
| 14 | 378 | 446 | SGVKPRRIRYNKTSHDYHLEEFEASLNMTPQTSIASGHETDPINHAYGTQADLLPYTRSSNITSTDTGS | 69 |
| 15 | 490 | 500 | ASFARRRRLGR | 11 |
| **NP_042022.1 matrix protein** | | | | |
| 1 | 7 | 11 | YVELK | 5 |
| 2 | 13 | 17 | KVIVP | 5 |
| 3 | 29 | 59 | VGGTSRNQFLNIPFLSVKEPLQLPREKKLTD | 31 |
| 4 | 90 | 97 | LSVYKDPI | 8 |
| 5 | 105 | 113 | LNKDQSKHA | 9 |
| 6 | 123 | 139 | YRLRNIGVGPLGPDIRS | 17 |
| **NP_042020.1 nucleoprotein** | | | | |
| 1 | 5 | 56 | RRLVDDADAMEDQDLYEPPASLPKLPGKFLQYTVGGSDPHPGIGHEKDIRQN | 52 |
| 2 | 58 | 70 | VALLDQSRRDMFH | 13 |
| 3 | 95 | 129 | VPRESYLSTPVTRGEQTVVKTAKFYGEKTTQRDLT | 35 |
| 4 | 153 | 164 | SKIKAGAEQIKK | 12 |
| 5 | 173 | 183 | LNRPSHGETAT | 11 |
| 6 | 185 | 199 | LQMFNPHEAIDWING | 15 |
| 7 | 215 | 224 | FESPGKEFMD | 10 |
| 8 | 262 | 280 | IRDFLEVSAKLKEDHADLF | 19 |
| 9 | 297 | 300 | RSFP | 4 |
| 10 | 309 | 355 | WSKKENPTMAGYRASTIQPGASVKETQLARYRRREISRGEDGAELSG | 47 |
| 11 | 365 | 366 | GV | 2 |
| **YP_009272535.1 X protein** | | | | |
| 1 | 17 | 84 | NGNATIESGRLPGGRRRSPDTTTGTTGVTKTTEGPKECIDPTSRPAPEGPQEEPLHDLRPRPANRKGA | 68 |
| **NP_042024.3 RNA-dependent RNA polymerase** | | | | |
| 1 | 7 | 19 | SSVRYALTNPRVR | 13 |
| 2 | 34 | 34 | Q | 1 |
| 3 | 36 | 50 | SYSREADIGPKRLGN | 15 |
| 4 | 99 | 105 | LWEQGSF | 7 |
| 5 | 144 | 152 | RHNVSVSSD | 9 |
| 6 | 210 | 221 | RVELETPQALLS | 12 |
| 7 | 239 | 256 | VSKNAKWPPVHLLPGCDK | 18 |
| 8 | 260 | 280 | NARELGRWSPAFDRRWQLFEK | 21 |
| 9 | 287 | 295 | ADLDMDPDF | 9 |
| 10 | 302 | 336 | KAIISSRRDWVFEYNAAAFWKKYGERLERPPARSG | 35 |
| 11 | 349 | 350 | LD | 2 |
| 12 | 361 | 366 | RGAVEF | 6 |
| 13 | 376 | 382 | KEKELKV | 7 |
| 14 | 413 | 427 | LKTHSMTMSSTALTH | 15 |
| 15 | 431 | 438 | RLSHTITK | 8 |
| 16 | 450 | 454 | SWCNG | 5 |
| 17 | 495 | 509 | FNPPYSLSGEPVEDG | 15 |
| 18 | 566 | 571 | QNNQLL | 6 |
| 19 | 585 | 593 | LAGHNLKVE | 9 |
| 20 | 595 | 595 | C | 1 |
| 21 | 609 | 614 | FFRGVP | 6 |
| 22 | 630 | 635 | GELFPN | 6 |
| 23 | 677 | 684 | PPAIMQDE | 8 |
| 24 | 733 | 734 | GV | 2 |
| 25 | 743 | 758 | VKLRIAPYPDWLSLVT | 16 |
| 26 | 761 | 774 | TSLNIAQVYRPERQ | 14 |
| 27 | 787 | 803 | SHSSRIATFFQQPLTEM | 17 |
| 28 | 816 | 816 | P | 1 |
| 29 | 852 | 863 | QAVHIEDVALES | 12 |
| 30 | 879 | 889 | EGYNMQPYLEG | 11 |
| 31 | 901 | 904 | LTWG | 4 |
| 32 | 916 | 929 | AEQFHPHSSVGAKA | 14 |
| 33 | 941 | 980 | QETLRSHHLTTRGDQPLYLGSNTAVKVQRGEITGLTKSRA | 40 |
| 34 | 995 | 1000 | KVRKVT | 6 |
| 35 | 1013 | 1049 | LEKGYTSDARPSIQGGTLTHRLPSRGDSRQGLTGYVN | 37 |
| 36 | 1063 | 1070 | HSFSKSSD | 8 |
| 37 | 1093 | 1098 | SGGVIS | 6 |
| 38 | 1111 | 1155 | FEKIDSEEFVLACEPQYRGAEWLISKPVTVPEQITDAEVEFDPCV | 45 |
| 39 | 1170 | 1199 | LVDIRASGHDIMEQRTWANLERFSVSDMQK | 30 |
| 40 | 1222 | 1227 | EKAGLI | 6 |
| 41 | 1235 | 1236 | GP | 2 |
| 42 | 1255 | 1270 | APLDRLSPRINFHSRG | 16 |
| 43 | 1284 | 1284 | P | 1 |
| 44 | 1286 | 1286 | I | 1 |
| 45 | 1291 | 1305 | VSGINSKYHAVSEAN | 15 |
| 46 | 1315 | 1338 | SVGVKPTQFVEETNDFTARGHHHG | 24 |
| 47 | 1344 | 1352 | WSKSRNQSQ | 9 |
| 48 | 1372 | 1376 | VDPAV | 5 |
| 49 | 1396 | 1404 | PAYYERLLE | 9 |
| 50 | 1408 | 1440 | CGAVSSRVDIPHSLAGRTHRGFAVGPDAGPGVI | 33 |
| 51 | 1454 | 1472 | CLEELEFNAYLDSELVDIS | 19 |
| 52 | 1517 | 1526 | LDIRPHLEEF | 10 |
| 53 | 1536 | 1538 | ILG | 3 |
| 54 | 1553 | 1589 | RKRPVLARHPWSADLKRITVGGRAPCPSAARLRDEDC | 37 |
| 55 | 1601 | 1604 | LTQL | 4 |

**Supplementary Table 3.** B-cells and T-cells overlapped epitopes in total.

| Start | End | Peptide | Length | Protective Antigen |
| --- | --- | --- | --- | --- |
| **NP_042023.1 glycoprotein** | | | | |
| 23 | 42 | FDLQGLSCNTDSTPGLIDLE | 20 | 1.3052 |
| 378 | 446 | SGVKPRRIRYNKTSHDYHLEEFEASLNMTPQTSIASGHETDPINHAYGTQADLLPYTRSSNITSTDTGS | 69 | 0.8235 |
| 178 | 194 | TLTYTPYILQSEVVNKT | 17 | 0.6892 |
| 490 | 500 | ASFARRRRLGR | 11 | 0.6356 |
| 206 | 227 | KIVSFDEFRRSYSLTNGSYQSS | 22 | 0.5366 |
| 238 | 256 | SSCRPRLKRRRRDTQQIEY | 19 | 0.4971 |
| 158 | 176 | CSTVSQQELESGKAMLSDG | 19 | 0.4774 |
| 106 | 116 | GCLNNSAPEDP | 11 | 0.1308 |
| 329 | 361 | ESTYYPPVDYNGRKYFLNDEGRLQTNTPEARPG | 33 | 0.0552 |
| 86 | 103 | GFFGSYSADRIINRYTGT | 18 | -0.0202 |
| **NP_042022.1 matrix protein** | | | | |
| 123 | 139 | YRLRNIGVGPLGPDIRS | 17 | 2.0186 |
| 29 | 59 | VGGTSRNQFLNIPFLSVKEPLQLPREKKLTD | 31 | 0.8414 |
| 105 | 113 | LNKDQSKHA | 9 | -0.229 |
| **NP_042020.1 nucleoprotein** | | | | |
| 309 | 355 | WSKKENPTMAGYRASTIQPGASVKETQLARYRRREISRGEDGAELSG | 47 | 0.7995 |
| 58 | 70 | VALLDQSRRDMFH | 13 | 0.7037 |
| 185 | 199 | LQMFNPHEAIDWING | 15 | 0.4952 |
| 262 | 280 | IRDFLEVSAKLKEDHADLF | 19 | 0.4724 |
| 95 | 129 | VPRESYLSTPVTRGEQTVVKTAKFYGEKTTQRDLT | 35 | 0.3641 |
| 5 | 56 | RRLVDDADAMEDQDLYEPPASLPKLPGKFLQYTVGGSDPHPGIGHEKDIRQN | 52 | 0.3407 |
| 153 | 164 | SKIKAGAEQIKK | 12 | 0.2738 |
| 173 | 183 | LNRPSHGETAT | 11 | -0.486 |
| 215 | 224 | FESPGKEFMD | 10 | -0.5652 |
| **YP_009272535.1 X protein** | | | | |
| 17 | 84 | NGNATIESGRLPGGRRRSPDTTTGTTGVTKTTEGPKECIDPTSRPAPEGPQEEPLHDLRPRPANRKGA | 68 | 0.5777 |
| **NP_042024.3 RNA-dependent RNA polymerase** | | | | |
| 36 | 50 | SYSREADIGPKRLGN | 15 | 1.6946 |
| 287 | 295 | ADLDMDPDF | 9 | 1.6857 |
| 743 | 758 | VKLRIAPYPDWLSLVT | 16 | 1.4869 |
| 852 | 863 | QAVHIEDVALES | 12 | 1.3262 |
| 1291 | 1305 | VSGINSKYHAVSEAN | 15 | 1.0398 |
| 1315 | 1338 | SVGVKPTQFVEETNDFTARGHHHG | 24 | 1.0205 |
| 1517 | 1526 | LDIRPHLEEF | 10 | 0.9929 |
| 1255 | 1270 | APLDRLSPRINFHSRG | 16 | 0.9882 |
| 1454 | 1472 | CLEELEFNAYLDSELVDIS | 19 | 0.9185 |
| 239 | 256 | VSKNAKWPPVHLLPGCDK | 18 | 0.8621 |
| 916 | 929 | AEQFHPHSSVGAKA | 14 | 0.8469 |
| 1013 | 1049 | LEKGYTSDARPSIQGGTLTHRLPSRGDSRQGLTGYVN | 37 | 0.7992 |
| 1553 | 1589 | RKRPVLARHPWSADLKRITVGGRAPCPSAARLRDEDC | 37 | 0.748 |
| 941 | 980 | QETLRSHHLTTRGDQPLYLGSNTAVKVQRGEITGLTKSRA | 40 | 0.7111 |
| 1408 | 1440 | CGAVSSRVDIPHSLAGRTHRGFAVGPDAGPGVI | 33 | 0.7082 |
| 495 | 509 | FNPPYSLSGEPVEDG | 15 | 0.6944 |
| 260 | 280 | NARELGRWSPAFDRRWQLFEK | 21 | 0.6166 |
| 585 | 593 | LAGHNLKVE | 9 | 0.6072 |
| 761 | 774 | TSLNIAQVYRPERQ | 14 | 0.586 |
| 7 | 19 | SSVRYALTNPRVR | 13 | 0.5252 |
| 879 | 889 | EGYNMQPYLEG | 11 | 0.5204 |
| 302 | 336 | KAIISSRRDWVFEYNAAAFWKKYGERLERPPARSG | 35 | 0.5199 |
| 413 | 427 | LKTHSMTMSSTALTH | 15 | 0.5142 |
| 1170 | 1199 | LVDIRASGHDIMEQRTWANLERFSVSDMQK | 30 | 0.5047 |
| 1111 | 1155 | FEKIDSEEFVLACEPQYRGAEWLISKPVTVPEQITDAEVEFDPCV | 45 | 0.4643 |
| 1344 | 1352 | WSKSRNQSQ | 9 | 0.4579 |
| 1396 | 1404 | PAYYERLLE | 9 | 0.3418 |
| 144 | 152 | RHNVSVSSD | 9 | 0.3122 |
| 210 | 221 | RVELETPQALLS | 12 | -0.3201 |
| 787 | 803 | SHSSRIATFFQQPLTEM | 17 | -0.2176 |

**Supplementary Table 4.** Results of physicochemical properties analysis.

| Physical and Chemical Properties | Result |
| --- | --- |
| Number of amino acids | 229 |
| Molecular weight | 23729.61 |
| Theoretical pI | 8.48 |
| Total number of negatively charged residues (Asp + Glu) | 22 |
| Total number of positively charged residues (Arg + Lys) | 25 |
| Formula | C_1032_H_1617_N_317_O_313_S_8_ |
| Total number of atoms | 3287 |
| Aliphatic index | 63.01 |
| Grand average of hydropathicity (GRAVY) | -0.614 |

**Supplementary Table 5.** Related results optimized using GalaxyRefine server.

| Model | GDT-HA | RMSD | MolProbity | Clash score | Poor rotamers | Rama favored |
| --- | --- | --- | --- | --- | --- | --- |
| Initial | 1.0000 | 0.000 | 2.393 | 21.1 | 0.0 | 89.0 |
| MODEL 1 | 0.9683 | 0.373 | 2.462 | 31.3 | 0.6 | 92.1 |
| MODEL 2 | 0.9727 | 0.367 | 2.462 | 31.3 | 0.6 | 92.1 |
| MODEL 3 | 0.9716 | 0.361 | 2.473 | 33.3 | 0.6 | 92.5 |
| MODEL 4 | 0.9640 | 0.387 | 2.474 | 32.1 | 0.6 | 92.1 |
| MODEL 5 | 0.9574 | 0.384 | 2.501 | 33.0 | 0.6 | 91.6 |
